# ﻿Synopsis of the genus *Elymus* (Poaceae) in Uzbekistan (Middle Asia) with a description of *Elymusuzbekistanicus* a new species from Turkestan Mts

**DOI:** 10.3897/phytokeys.257.142950

**Published:** 2025-06-03

**Authors:** Kumush B. Alieva, Yilong Peng, Adilet Usupbaev, Komiljon Sh. Tojibaev, Ziyoviddin Yusupov, Ibrokhimjon Ergashov, Dilnoza Azimova, Zhilin Jiang

**Affiliations:** 1 Institute of Botany, Academy of Sciences of Uzbekistan, Tashkent 100125, Uzbekistan Institute of Botany, Academy of Sciences of Uzbekistan Tashkent Uzbekistan; 2 Faculty of Science, St Lucia Campus, The University of Queensland Brisbane, QLD, 4072, Australia The University of Queensland Brisbane QLD Australia; 3 Institute of Biology, National Academy of Science of the Kyrgyz Republic, Bishkek, 720071, Kyrgyzstan Institute of Biology, National Academy of Science of the Kyrgyz Republic Bishkek Kyrgyzstan; 4 Faculty of Natural Sciences, Fergana State University, Fergana, Uzbekistan Fergana State University Fergana Uzbekistan; 5 Jizzakh State Pedagogical University, Jizzakh, 130100, Uzbekistan Jizzakh State Pedagogical University Jizzakh Uzbekistan; 6 Institute of Agricultural and Garden Technology, Puer University, Puer, Yunnan, China Puer University Puer China

**Keywords:** *Anthosachne*, *Elymus*, new species, phylogeny, taxonomy, Turkestan Range

## Abstract

A synopsis of the genus *Elymus* in Uzbekistan, including a description of the genus and species, a key to species identification, illustrations, geographical distribution, and habitat requirements, are presented in detail. According to molecular analyses, including chloroplast and nuclear DNA data, the species forms a distinct clade with members of sect. Anthosachne. Morphologically, it is distinguished by significantly higher pubescence compared to other related species. As a result of our morphological and molecular evidence, a new species *Elymusuzbekistanicus* is described from the Turkestan Mts (West Pamir-Alay). It differs from the closely related *E.praeruptus* Tzvelev in that the upper surface of the leaf blades is densely covered with short hairs along rather prominent ribs. The axis segments of rather loose spikes are usually smooth or with scattered short spines along the ribs.

## ﻿Introduction

The Republic of Uzbekistan is a Middle Asian country that is part of the Tethyan floristic subkingdom of the Holarctic kingdom, which lies in the Irano-Turanian region (Takhtajan 1986; Tojibaev et al. 2016; Pimenov et al. 2023). In Uzbekistan, floristic surveys began over 150 years ago (Pimenov et al. 2023). The initial six-volume edition of the Flora of Uzbekistan provided the first overview of the diversity of vascular plant species in the country (Schreder, Korovin, 1941; Vvedensky, Korovin, 1953; Vvedensky, Korovin, 1955; Vvedensky, 1959; Vvedensky, 1961; Vvedensky, 1962). In accordance with Tojibaev et al. (2019), the flora of Uzbekistan includes 4,148 vascular plant species, comprising 485 naturalized, alien, and cultivated taxa and 3,663 native species. Since 2021, several papers on new species for science and new records to the flora of Uzbekistan have been published (Juramurodov et al. 2021; Sennikov et al. 2022; Shchegoleva et al. 2022; Khassanov et al. 2023; Pimenov et al. 2023; Alieva et al. 2024b; German et al. 2024; Levichev et al. 2025). In addition, recently published taxonomic revisions have significantly enhanced our understanding of the taxonomy and distribution of species and genera in Uzbekistan, including *Eremurus* M.Bieb (Makhmudjanov et al. 2022), *Tulipa* L. (Tojibaev et al. 2022), *Salvia* L. (Turdiboev et al. 2022), *Iris* L. (Sennikov et al. 2023), *Hedysarum* L. (Juramurodov et al. 2024), and others. A comprehensive inventory of the national vascular plants is part of a new initiative called “Flora of Uzbekistan”, which eventually seeks to produce a comprehensive standard Flora based on contemporary standards and guidelines (Sennikov et al. 2016). The first six volumes of Flora of Uzbekistan’s second edition are now available (Sennikov 2016, 2017, 2019, 2022, 2023a, 2023b). Treatments for other families are also undertaken (Pimenov et al. 2023). The revision of some polymorphic families in Uzbekistan, notably Poaceae, is now underway.

The family Poaceae is among the most diverse and ecologically important plant families, encompassing more than 789 recognized genera (Soreng et al. 2022). They also belong to one of the most species-rich families in Middle Asia as well as in Uzbekistan (Schreder and Korovin 1941; Tzvelev 1976). *Elymus* L. (Poaceae), with more than 150 perennial species of temperate and subtropical regions (Lu 1994; Frawley et al. 2019; Govaerts 2024), is one of the largest genera of Triticeae (Soreng et al. 2022; Yadav et al. 2023), and together with *Poa* L., *Stipa* L., *Festuca* Tourn. Ex L., and *Bromus* L. belong to the species-rich genera of grasses in Uzbekistan (Nikiforova 1968, Tzvelev 1976, Nobis et al. 2020). More than 36 species of *Elymus* were described from 1968 to 1976, primarily by Tzvelev (1968, 1970, 1972, 1973, 1976), whereas Nikiforova (1968) listed 25 species in Asiae Media. Based on newly available information, 24 of them are recognized as species of *Campeiostachys* Drobow, *Leymus* Hochst., or *Psathyrostachys* Nevski ex Roshev. (Soreng et al. 2017). According to the POWO (2025), 21 species of *Elymus* are recognized in Middle Asia with 7 being regional endemics (Govaerts 2024). In accordance with Tzvelev (1976), there are no endemic species to Uzbekistan, although two species, *E.praeruptus* Tzvelev and *E.macrochaetus* (Nevski) Tzvelev are Middle Asian endemics.

The most significant source of data for plant taxonomic research is herbaria (Gaem et al. 2024). Taxonomic decision-making in difficult plant groupings can be accelerated with the use of modern tools and resources, such as digital collections and herbarium DNA sequencing (Gaem et al. 2024). Since its establishment in 1831–1835, the National Herbarium of Uzbekistan (TASH), Institute of Botany, Academy of Sciences of the Republic of Uzbekistan, has accumulated more than 1.6 million herbarium specimens, making it the largest herbarium collection of Middle Asian plant species in the world. As a result, this collection has become one of great importance for the study of the diversity of plants in Uzbekistan and its surroundings and for the creation of a digital database. There are more than 38,000 herbarium specimens of the Poaceae family preserved in the TASH. Intensive research on Poaceae, including *Elymus*, in Middle Asia has led to significant findings. According to the TASH, several new species of *Elymus*, including *E.macrochaetus* and *E.glaucissimus*, were discovered in Uzbekistan and Kyrgyzstan, respectively (Alieva et al. 2024b). Here we report the discovery of another undescribed species of *Elymus* from the Turkestan Range of Uzbekistan. As a result of analyzing herbarium materials stored in TASH, specimens of *Elymus* different from known species were discovered. A comprehensive revision of *Elymus* in Uzbekistan is necessary due to taxonomic changes that have occurred over the past 85 years. Recent research, particularly the Flora of Uzbekistan project (Sennikov et al. 2016), has significantly advanced our understanding of flora of Uzbekistan. Special attention has been given to nomenclature, typification, and detailed mapping of the distributions of species.

In this study, we revised all specimens representing the genus *Elymus* collected in Uzbekistan and reconstructed the phylogenetic relationships of the *Elymus* members using ITS and whole chloroplast genome data. This analysis has enabled us to: 1) present the first synopsis of the genus *Elymus* in the flora of Uzbekistan, considering all nomenclatural changes, providing descriptions of the genus and species, a key to species identification, illustrations, remarks on their general distribution (with map), habitat, phenology, and 2) as a result of the comprehensive study of the *Elymus* herbarium specimens stored in the TASH herbarium collection, the discovery of a new species and the diagnosis of its morphological and molecular traits.

## ﻿Material and methods

### ﻿Sampling and morphological analyses

The study was based on 142 herbarium specimens of *Elymus* species from the TASH and more than 40 specimens from the herbarium collections of Samarkand State University (SAMDU), as well as virtual herbarium data from MW, US, USDA-NPGS, and W [acronyms according to Thiers (2021)]. Additionally, online resources such as Plantarium (https://www.plantarium.ru/), GBIF (https://www.gbif.org/), and JACQ Virtual Herbarium (https://www.jacq.org/) were used to support the study (Alieva 2023; Alieva et al. 2024a). The list of species and specimens examined is presented in subchapters related with geographical distribution of particular species presented below.

Morphological measurements of the spikes, spikelets, glumes, stems, nodes, ligules, lemmas, and paleas were taken from these four herbarium specimens, as well as from ten additional species of *Elymus*, using a binocular microscope (Bresser Advance ICD 10x-160x Zoom Stereo-Microscope). Five diagnostic features, leaf blades, spikelet axis, lemma, palea, and anthers, were analyzed and compared with species closely related to *E.nevskii* Tzvelev and *E.praeruptus* Tzvelev, using specimens in TASH, SAMDU, MW, US, USDA-NPGS, and W (herbarium abbreviations according to Thiers, 2021). We also referred to descriptions of morphological characters presented in the Flora of Turkmenistan (Fedtschenko 1932), Flora of Uzbekistan (Drobow 1941), Flora of Kyrgyzstan (Rozhevits 1950), Flora of Kazakhstan (Kuznetsov 1956), Flora of Tajikistan (Sidorenko 1957), and Flora Iranica (Bor 1970), along with additional scientific articles (Salomon 1990; Barkworth et al. 1996; Pusalkar et al. 2008; Jacobs and Barkworth 2009; Yadav et al. 2023).

Geographic regionalization of the study area was based in accordance with Tojibaev et al. (2016). We georeferenced the locations of historical herbarium specimens using Google Earth software (https://earth.google.com/web/).

### ﻿Taxon sampling for phylogenetic analysis

For phylogenetic analysis, we used newly generated nuclear ribosomal DNA (nrDNA) and chloroplast genome sequences for the undescribed species of *Elymus* along with sequences from additional species of *Elymus* distributed in Uzbekistan, including *E.caninus* (L.) L., *E.lachnophyllus* (Ovcz. & Sidorenko) Tzvelev, *E.fedtschenkoi* Tzvelev, *E.longearistatus* (Boiss.) Tzvelev, *E.macrochaetus* (Nevski) Tzvelev, *E.nevskii* (a synonym of *E.dentatus* (Hook.f.) Tzvelev; identity of the species was based on the taxonomic treatments of Tzvelev 1976, Tzvelev and Probatova 2019), *E.praeruptus*, *E.transhyrcanus* (Nevski) Tzvelev, *E.tschimganicus* (Drobow) Tzvelev, and *E.tianschanigenus* (Drobow) Czerep. Sequences from five additional species of *Elymus* available in GenBank (www.ncbi.nlm.nih.gov/Genbank) were included in the analysis (Appendix 1). Based on Dong et al. (2015), we used *Bromusinermis* Leyss. as an outgroup to determine the systematic position of the undescribed species. Accession numbers for all sequences used in this study are provided in Appendix 1.

### ﻿Extraction of DNA, assembly of sequences, and annotation

In accordance with the manufacturer’s instructions, DNA isolation was carried out using a Plant Genomic DNA Kit (TIENGEN Biotech, Beijing, China). Using the Genomic DNA Sample Prep Kit (Illumina) and the manufacturer’s instructions, DNA was sheared to create a 350-bp (insert size) sequence library. The Illumina HiSeq 4000 at Beijing Novogene Bioinformatics Technology Co., Ltd, Beijing, China was used to sequence the sample using 150 paired-end reads. The Next Generation Sequencing (NGS) QC Tool Kit with default settings was utilized for raw data processing (Patel and Jain 2012). Using the genome of *Elymussibiricus* L. NC_058919 (Xiong et al. 2023) as a reference, the resulting clean reads were assembled in NovoPlasty v.3.8.3 (Dierckxsens et al. 2017). The software Geneious v.10.0.2 was used to sort and merge contigs produced by NovoPlasty into a single draft sequence and for gene annotations (Kearse et al. 2012). The assembly of the nrDNA sequencing reads was done with GetOrganelle v.1.7.4.1 (Jin et al. 2020).

### ﻿Phylogenetic analyses

The MAFFT v.7.311 function (Katoh and Standley 2013) in Geneious v.10.0.2 software (Kearse et al. 2012) was used for alignment. MEGA X software (Kumar et al. 2018) was used to manually align the data when necessary. Due to differences in the positions of the undescribed species in the nuclear and plastid trees, phylogenetic reconstructions were carried out using the nuclear and plastid data independently. FigTree v1.4.0 (Rambaut 2012) was used to visualize trees. Maximum likelihood (ML), maximum parsimony (MP), and Bayesian inference (BI) were used to reconstruct the phylogenetic trees. For ML, we utilized raxmlGUI 2.0 (Edler et al. 2020) with 1000 bootstrap replicates, and for BI, we used MrBayes v.3.1.2 (Huelsenbeck and Ronquist 2001). A model of nucleotide substitutions was chosen for analysis using jModelTest2 on XSEDE (www.phylo.org) in accordance with the Akaike Information Criterion (AIC). Phylogenetic studies were also carried out with the maximum parsimony (MP) method using PAUP*4.0a169 (Swofford 2002). The MP and ML bootstrap percentages were labelled on their respective branches of the BI tree.

## ﻿Results

**Figure 1.**
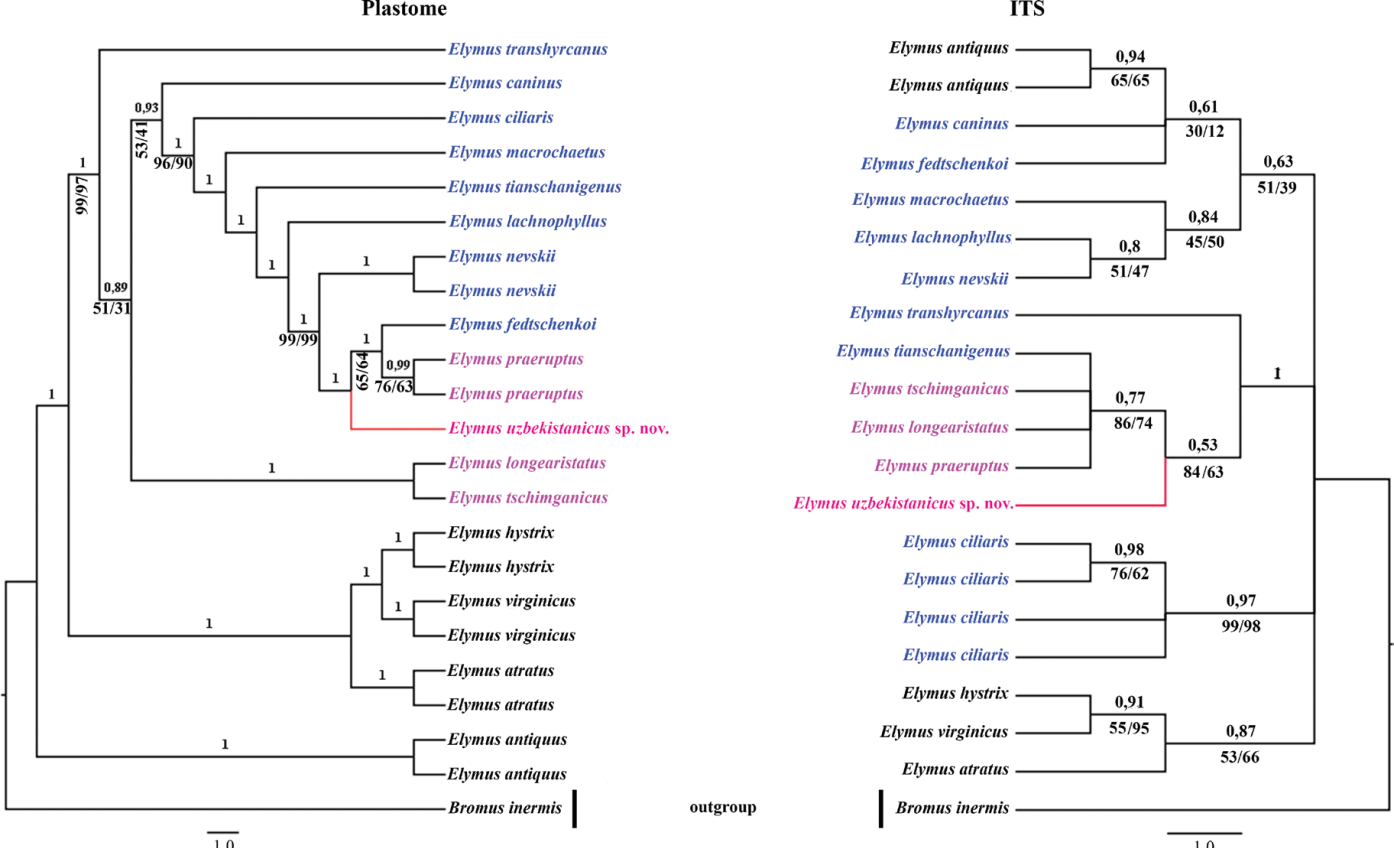
Plastome (whole cpDNA genome) and ITS-based trees showing phylogenetic position of *Elymusuzbekistanicus* sp. nov. in sect. Anthosachne. Pink represents species of sect. Anthosachne, while blue illustrates species of sect. Goulardia (Husn.) Tzvelev (Tzvelev 1970). Bayesian posterior probability (PP) is displayed on each branch, while maximum likelihood (ML) and maximum parsimony (MP) values are shown below branches, respectively. Full results (100%) of ML and MP analyses are not shown.

According to the phylogenetic trees, all representatives of *Elymus* recorded in Uzbekistan, form a common clade, indicating a shared chloroplast genomic origin. This suggests that sect. Anthosachne and sect. Goulardia to which belong analyzed species have a common evolutionary history in terms of plastid inheritance, despite their potential morphological and nuclear genomic differences. However each of the species used in the analyses are well supported and represent independent entity within the genus (Fig. 1). More over, the analysis reveals the existance of a new species, here and below names as *Elymusuzbekistanicus* (Fig. 1). It forms a separate clade in both the nrDNA and cpDNA trees, indicating that it is more genetically distinct than the other species. In the plastid tree, *E.uzbekistanicus*, *E.praeruptus*, and *E.fedtschenkoi* formed a well-supported clade (PP = 1, MP and ML = 100%). The nuclear tree based on ITS sequence confirmed a close relationship among *E.uzbekistanicus* and other species of sect. Anthosachne, such as *E.praeruptus*, *E.longearistatus* and *E.tschimganicus* (Tzvelev 1973), which means *E.uzbekistanicus* is sister to them (PP = 0.53 ML = 84%, and MP = 63%).

The representatives of the genus *Elymus* were found in five (31.25%) of the sixteen botanical-geographical districts in Uzbekistan. Eleven species were found in the Pamir-Alay and Tian Shan mountain ranges, what makes them the most abundant in *Elymus* species (Table 2). However, the most species-diverse area shows itself to be the Pamir-Alay mountain system—10 species distributed in the Hissar, Alay, Kuhitang, Malguzar, and Turkestan ranges. In the Western Tien Shan mountain system, including the Chatkal, Kurama, Maidantal, Karzhantau, Pskem, and Ugom ranges seven species were confirmed.

### ﻿Synopsis of the genus *Elymus* in Uzbekistan

#### ﻿Description of the genus *Elymus*

Perennial plants (15) 20–150 (200) cm tall, usually forming more or less dense tussocks without creeping underground shoots. Stems erect, ranging from glabrous and smooth to rough and hairy. Leaf blades 1.2–15 (18) mm wide, flat or involute, glabrous or hairy. Leaf sheaths of vegetative shoots often closed up to ½ of their length from the base, with the upper part lanceolate; stem leaf sheaths almost split to the base, with or without ligule. Ligule 0.1–1.5 (2) mm long, often minutely ciliate along the edge. Inflorescence — a straight or drooping spike (3) 4–18 (30) cm long, with a persistent rachis that does not disarticulate at maturity. Spikelets arranged singly along the spike axis, not always in regular longitudinal rows, nearly sessile (with pedicels 0.3–1.5 mm long), all similar in size, (8) 10–25 (35) mm long, containing (2) 3–7 (11) bisexual flowers. Spikelet rachis rough or short-hairy, with well-developed articulation below each flower. Glumes range from lanceolate-ovate to narrowly lanceolate, unequal, (3) 4–14 (18) mm long (excluding awns), usually glabrous, more or less rough along the veins, with an acute or obtuse apex, often bearing an awn up to 8 (10) mm long. Lemmas 7–15 (17) mm long (excluding awns), lanceolate-elongate, leathery, smooth, rough, or short-hairy at the apex, sharp, often with a straight or slightly curved awn up to 50 (70) mm long or sometimes awnless. Callus rather long, bluntly triangular, usually with very short hairs. Palea slightly shorter than the lemma, more or less rough or ciliate along the keels. Stamens — 3, with anthers (0.7) 1–4 (5) mm long. Caryopses 5–9 mm long. Chromosomes large, n = 7 (Tzvelev 1976).

### ﻿Keys to the species of *Elymus* in Uzbekistan

#### ﻿Identification key to sections of *Elymus* in Uzbekistan

**Table:** 

1	Spikes with more than seven spikelets	**Elymussect.Anthosachne (Steud.) Tzvelev**
–	Spikes are relatively few in number, usually with only 3–7 spikelets	**Elymussect.Goulardia (Husn.) Tzvelev**

##### ﻿ Elymus sect. Anthosachne

Taxon classificationPlantaePoalesPoaceae

(Steud.) Tzvelev in Novosti Sist. Vyssh. Rast. 10: 25 (1973)

DCFA0342-EC7E-5C3A-96E3-EBE72D34A0F9

###### Type.

*Elymusaustralasicus* (Steud.) Tzvelev (= *Anthosachneaustralasica* Steud.).

###### Description.

Spikes usually drooping, with relatively few (often only 3–7) spikelets (solitary in two regular longitudinal rows). Glumes 4–12 mm. Awn of lemma more or less bent to the side 17–50 mm.

### ﻿Key to species of Elymussect.Anthosachne in Uzbekistan

**Table:** 

1	Spikes erect, less often slightly drooping; lower glumes less than 1.5 times shorter than the adjacent lemmas (not including awn)	**2**
–	Spikes drooping; lower glumes 1.5–3 times shorter than the adjacent lemmas (not including awn)	**3**
2	Internodes of spikes with long hairs over the entire surface; leaf blades with protruding long, dense hairy on both surfaces; stems and nodes densely long hairy	**11** (***E.uzbekistanicus*)**
–	Internodes of spikes along lateral ribs glabrescent or covered with spines; leaf blades only adaxially with short or scattered hairs or glabrescent; stems and nodes usually glabrous or glabrescent	**7 (*E.praeruptus*)**
3	Awn of lemmas 35–60(70) mm; anthers 3–4(4.7) mm; internodes of spike axis noticeably elongate	**4 (*E.longearistatus*)**
–	Awn of lemmas up to 30(35) mm; anthers 2–3 mm; internodes of spikes axis shorter	**10 (*E.tschimganicus*)**

#### ﻿ Elymus sect. Goulardia

Taxon classificationPlantaePoalesPoaceae

(Husn.) Tzvelev in Spisok Rast. Gerb. Fl. S.S.S.R. Bot. Inst. Vsesojuzn. Akad. Nauk 18: 27 (1970). Type: Elymus caninus (L.) L.

D8EB0A0B-474E-5069-A578-A7F549EB052F

≡ Agropyronsect.Goulardia (Husn.) Holmb. in Scand. Fl. 2: 269 (1926).
≡ Goulardia Husn., Gram. 83 (1896).

##### Description.

Spikes straight or slightly drooping, with a fairly large number of spikelets (solitary in two regular longitudinal rows). Glumes (4) 5–15 (18) mm. Lemma either awned or awnless. Leaf blades usually flat, green.

### ﻿Key to species of Elymussect.Goulardia in Uzbekistan

**Table:** 

1	Lemmas of all florets in spikelet awnless or with awn to 6 mm	**2**
–	Lemmas of all or only upper florets in spikelet with awn more than 6 mm	**4**
2	Lemmas glabrous or glabrescent over almost entire abaxial surface, sometimes short-hairy at base and often more or less rough only near apex; anthers 3.5–4.5 mm	**9 (*E.transhyrcanus*)**
–	Lemma entirely or almost entirely with dense spaced spines or hairs abaxially; anthers 1.5–2.5 mm	**3**
3	Nodes of stems always and abaxial surface of leaf blades usually glabrous	**6 (*E.nevskii*)**
–	Nodes of stem and both surface of leaf blades short hairy	**3 (*E.lachnophyllus*)**
4	Awn of lemmas straight or often slightly sinuous	**5**
–	Awn of lemmas more or less bent to side	**2 (*E.fedtschenkoi*)**
5	Lemmas abaxially partly glabrous or glabrescent	**1 (*E.caninus*)**
–	Lemmas abaxially entirely with spines or hairs	**6**
6	Anthers to 3 mm; awn of lemmas 6–15 mm	**8 (*E.tianschanigenus*)**
–	Anthers 3–4.5 (5) mm; awn of lemmas 15–30 (35) mm	**5 (*E.macrochaetus*)**

#### ﻿1. Elymus caninus

Taxon classificationPlantaePoalesPoaceae

(L.) L. in Fl. Suec., ed. 2: 39 (1755)

E6771FDA-A543-5760-AF54-0B79B6D7ADED

≡ Agropyroncaninum (L.) P.Beauv. in Ess. Agrostogr.: 102 (1812). ≡ Elytrigiacanina (L.) Drobow in Fl. Uzbekistan. 1: 539 (1941). ≡ Goulardiacanina (L.) Husn. in Graminées: 83 (1899). ≡ Roegneriacanina (L.) Nevski in Trudy Bot. Inst. Akad. Nauk S.S.S.R., Ser. 1, Fl. Sist. Vyssh. Rast. 1: 24 (1933). ≡ Triticumcaninum L. in Sp. Pl.: 86 (1753), nom. cons.
= Agropyronabchazicum Woronow in Vĕstn. Tiflissk. Bot. Sada 22: 2 (1912). Type. ABKHAZIA. Hab. in pratis lapidosis subalpinis montis Dzychscha (Abchaziae et Circassiae confines) 6500', ubi 4 (17). VIII. 1905 detexi. (It lives in the stony meadows of the subalpine mountain Dzychscha (borders of Abkhazia and Circassia) 6500', 4 (17) August 1905. Collectorunknown) (holotype LE).
= Roegneriatuskaulensis Vassilcz. in Bot. Mater. Gerb. Bot. Inst. Komarova Akad. Nauk S.S.S.R. 15: 36 (1953). Type. KYRGYZSTAN. Middle Asia, river basin Khoja-Ata, territory of the Arkit forestry, Sai, south of Sarai-Sai, at the side of the floodplain, 1500 m, 21 August 1950, *I.T. Vasilchenko* s.n. (holotype LE).

##### Type.

Europe • Herb. Linnaeus No. 100.9 (LINN), typ. cons. prop. (designated by Andrés Sánchez et al. 2021: 1138).

##### Description.

**Figure 2.**
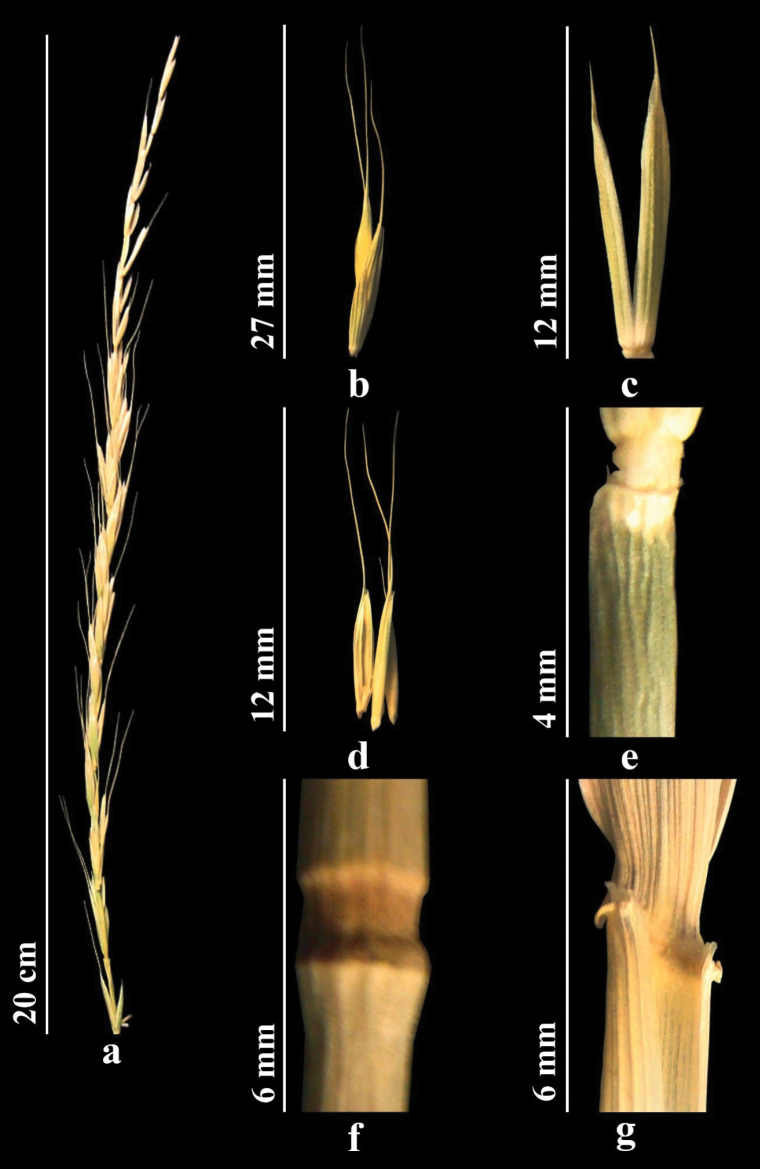
*Elymuscaninus***a** spike **b** spikelet **c** glumes **d** lemmas and paleas **e** stem **f** node **g** ligule (https://doi.org/10.13140/RG.2.2.12460.27527).

Stems 75–150 cm, glabrous, smooth. Leaves 5–10 mm wide, flat, green or bluish green, rough, glabrous or sparsely hairy abaxially. Spikes straight or slightly drooping, with a fairly large number of spikelets. Spikelets solitary along axis of the spike. Internodes of spikelet with short hairs. Glumes lanceolate, abruptly narrowed apically, usually short awned; awn to 2 mm. Lemma abaxially mostly glabrous, smooth, with spicules only apically; awn straight or often slightly curved, 1.5–1.8 cm. Palea with small, densely arranged spicules along keels, apex narrowly rounded. Anthers to 3 mm (Fig. 2). *2n* = *28.* (Tzvelev and Probatova 2019).

##### Phenology.

Flowering and fruiting: June-August.

##### Habitat.

In forests (*Abies*, *Picea*, *Corylus*, *Juniperus*, stunted *Juniperus*, tugai, and open forests), shrubs (polydominant, mesophytic deciduous), and meadows (floodplains, mesophytic), across all mountain belts, 700–2800 m.

##### General distribution.

North America, Europe, Scandinavia, the Mediterranean, Turkey, the Caucasus, Western and Eastern Siberia, Iran, China (Kashgar), Middle Asia (Tarbagatai, Dzhungarian Alatau, Tian Shan, Aral-Caspian Lowland).

##### Distribution in Uzbekistan.

**Figure 3.**
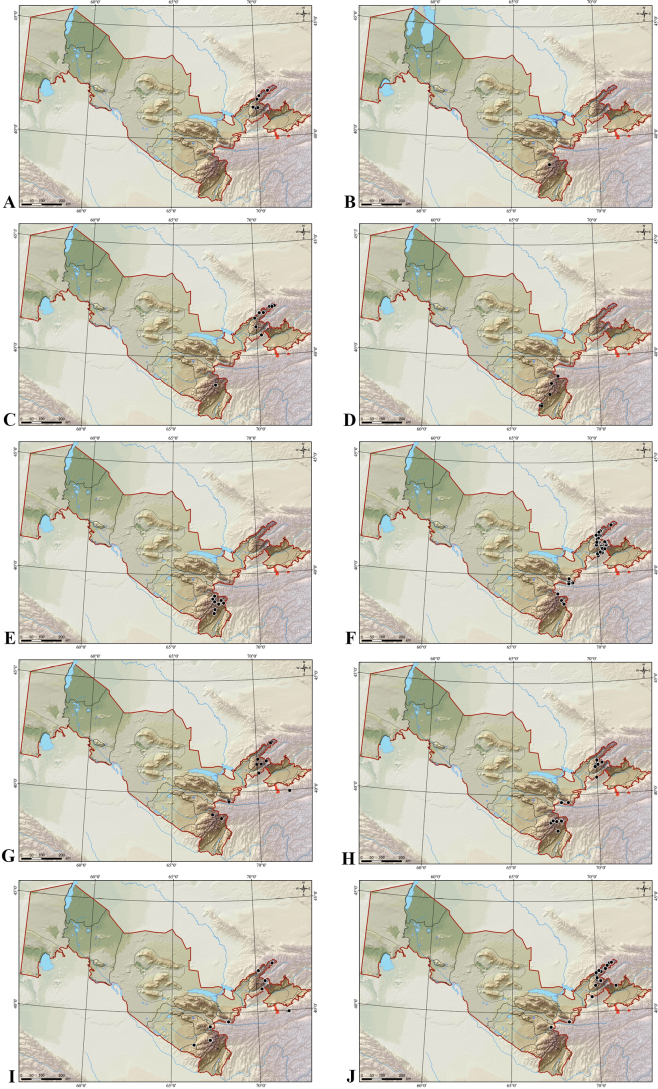
Distribution maps of *Elymus* species in Uzbekistan **A***E.caninus***B***E.lachnophyllus***C***E.fedtschenkoi***D***E.longearistatus***E***E.macrochaetus***F***E.nevskii***G***E.praeruptus***H***E.transhyrcanus***I***E.tschimganicus***J***E.tianschanigenus*.

Tashkent Region (Fig. 3A). I-1 **Western Tian Shan district**. I-1-a **Ugam-Pskem region.** UGAM RANGE (Bostanlyk, eastern side along the bank of the stream in the upper reaches of the Navali-sai gorge, 2800 m, 30.07.1953, *Pavlov 480* [MW0808430, MW0808431; MW0808432]; In the vicinity of Sidzhak village, Sidzhak-sai stream. Alt.: 800–1000 m, 24.07.1973, *Vašák* s.n. [W1981–0007920]; Asia centralis, Tian-shan, montes Ugamski khrebet, in vicinitate pagi Sidzhak, apud rivulum Sharkrama-sai. Alt.: 700–1000 m, 26.07.1973, *Vašák* s.n. [W1982–0003370]); MAYDANTAL RANGE (Oygaing river valley, 2 km northeast of the mouth of the right bank of the Beshtor river. h=1650–1700 m. Birch Grove, 22.08.1971, *Puchkova, Blativitch 45* [TASH040340!, TASH040341!, TASH040342!]); I-1-b **Western Chatkal region**. CHATKAL RANGE (Gorge of the Kashka-Su River, 26.07.1936, *Korotkova, Titov 1691* [TASH040338!]; New section of Reserve, Chatkal Mountains, 70 km E of Tashkent, *314205* [USDA_NPGS3389682], *314210* [USDA_NPGS3389676]; Parkent nature reserve, 08.08.1960, *Khokhrjakov* s.n [W1963–0014241]).

#### ﻿2. Elymus fedtschenkoi

Taxon classificationPlantaePoalesPoaceae

Tzvelev in Novosti Sist. Vyssh. Rast. 10: 21 (1973)

F7952899-FDA3-52D5-9697-514E2A261564

≡ Roegneriafedtschenkoi (Tzvelev) N.R.Cui in Claves Pl. Xinjiang. 1: 158 (1982), nom. superfl.
= Agropyroncurvatum Nevski in Izv. Bot. Sada Akad. Nauk S.S.S.R. 30: 629 (1931 publ. 1932). — non Elymuscurvatus (Nevski) D.F.Cui in Fl. Xinjiangensis 6: 197 (1996), nom. illeg.; ≡ Roegneriacurvata (Nevski) Nevski in Trudy Sredne-Aziatsk. Gosud. Univ., Ser. 8b, Bot. 17: 67 (1934).
= Agropyronmacrolepis Drobow in Repert. Spec. Nov. Regni Veg. 21: 41 (1925). Type. Uzbekistan. Prov. Syr-darja. Distr. Aulie-ata. Fl. Arabik. (*Abolin et Popov 8741*, 1921); ≡ Elymusmacrolepis (Drobow) Tzvelev in Trudy Bot. Inst. Komarova Akad. Nauk SSSR, Rast. Tsentral. Azii 4: 217 (1968). Type. Kazakhstan. Northern Tian Shan, Ketmen Range (holotype LE).

##### Type.

Kazakhstan (Northern Tian Shan) • Northern slope of the Ketmen Mountains, Kyrgyzsay Gorge, Podgorny settlement, 19 July 1910, *A. Michelson* s.n. (holotype LE).

##### Description.

**Figure 4.**
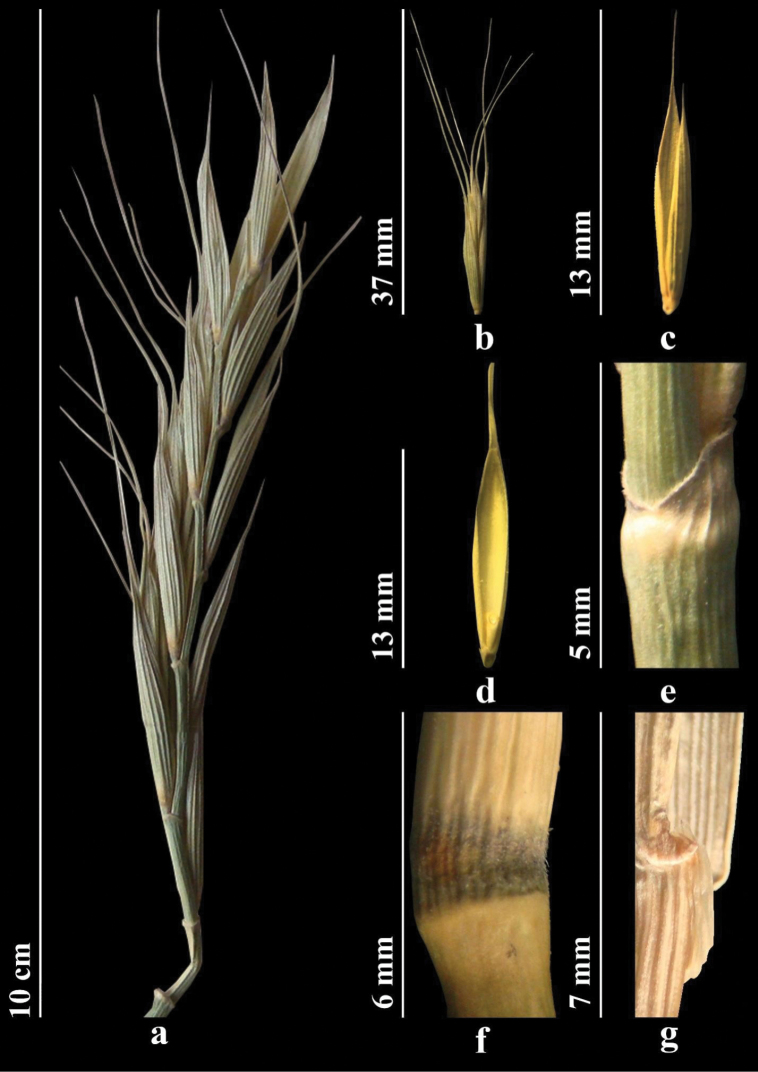
*Elymusfedtschenkoi***a** spike **b** spikelet **c** glumes **d** lemmas and paleas **e** stem **f**node **g** ligule (https://doi.org/10.13140/RG.2.2.19171.16163).

Stems 50–100 cm, strong, straight, or slightly bent at base. Leaves 3–10 (12) mm wide, usually flat, green, adaxially glabrous or with scattered rather long hairs along slightly prominent ribs. Sheaths either glabrous or hairy. Ligule 1 mm. Spikes, usually green, straight or slightly drooping, often slightly one-sided, 7–16 cm. Spikelets appressed against axis of spike, 7–20 mm. Glumes broadly lanceolate, acute to acuminate. Lemma abaxially densely and evenly covered with fine spines transitioning into hairs; awn 20–40 mm. Palea lanceolate, obtuse, bristly along keels. Anthers 2–4 mm (Fig. 4). *2n* = *28* (Tzvelev and Probatova 2019).

##### Phenology.

Flowering and fruiting: June-August.

##### Habitat.

In meadows (floodplains), rocky communities (rocky-gravelly scree, cliffs), and gravel, in the middle, subalpine, and alpine mountain belts, 1100–4200 m.

##### General distribution.

Russia (Western Siberia, Altai), Afghanistan, Pakistan, China (Kashgar), Western Himalayas, Mongolia, Middle Asia (Tarbagatai, Dzungarian Alatau, Tian Shan, Pamir-Alay: Hissar, Darvaz, and Pamir Mountain ranges): Kazakhstan, Kyrgyzstan, Tajikistan, Uzbekistan.

##### Distribution in Uzbekistan.

Surkhandarya and Tashkent regions (Fig. 3C). I-1 **Western Tian Shan district.** I-1-a **Ugam-Pskem region.** KARZHANTAU RANGE (Syr-Darya region, Tashkent Gorge. The surroundings of Khumsan mountains Kerzhentau in the stones, high, 24.07.1922, *Simonova 285* [TASH040946!]); UGAM RANGE (Valley of the Pskem River. Upper reaches of Tepar-say, 17.08.1928, *Kultiasov 717* [TASH055306!]); PSKEM RANGE (Valley of the Pskem River, 28.08.1928, *Kultiasov 904* [TASH055305!]; River basin Pskem. River valley Oygaing, Description No. 41, 1941, *Momotov* s.n. [TASH040968!]; the upper reaches of the river Oygaing. Pass (4200 m) in the upper reaches of the Kzyl – Tor, 19.08.1956, *Zukerwanik 1515* [TASH040965!, TASH040970!]; Bostandyk district, upper reaches of the Barkrak-say gorge. On the southern rocky slope near the GRP base, 3300 m, 08.08.1959, *Pavlov 19a* [MW0808635]); MAYDANTAL RANGE (lower reaches of the Ayutor River, h=2120, 13.08.2021, *Maltsev* s.n. [TASH131348!]); I-1-b **Western Chatkal region**. CHATKAL RANGE (Mountains Tashkent Alatau. Kizyl-Nura Mountain. The upper reaches of the Parkent-say. Rubble slope, 09.08.1953, *Mailun, Nabiev 1139* [TASH040941!]); I-1-d **Кurama region.** KURAMA RANGE (Northern slopes. Upper reaches of the Lyashkarak-say. Subalpine belt. Tugai-sedge vegetation, 06.08.1939, *Kudryashev 1055* [TASH040942!]. I-7 **Hissar-Darvaz district.** I-7-a **Sangardak-Tupalang region.** HISSAR RANGE (Pamiro-Alay. Choriogul Mountains. Side slope, 7.08.1941, *Lopotiy, Pinhasov 77* [TASH040951!]).

#### ﻿3. Elymus lachnophyllus

Taxon classificationPlantaePoalesPoaceae

(Ovcz. & Sidorenko) Tzvelev in Novosti Sist. Vyssh. Rast. 9: 61 (1972)

6DAA97F1-07F4-59FC-9BBE-0B1B0C49D688

≡ Agropyronlachnophyllum (Ovcz. & Sidorenko) Bondarenko in Opred. Rast. Sred. Azii 1: 173 (1968). ≡ Roegnerialachnophylla Ovcz. & Sidorenko in Fl. Tadzhiksk. S.S.R. 1: 505 (1957).

##### Type.

Tajikistan • Kussavli-Saj, in juniperetis stepposis ad 2500–2600 m. Date unknown, *Ovczinnikov* s.n. (holotype LE).

##### Description.

**Figure 5.**
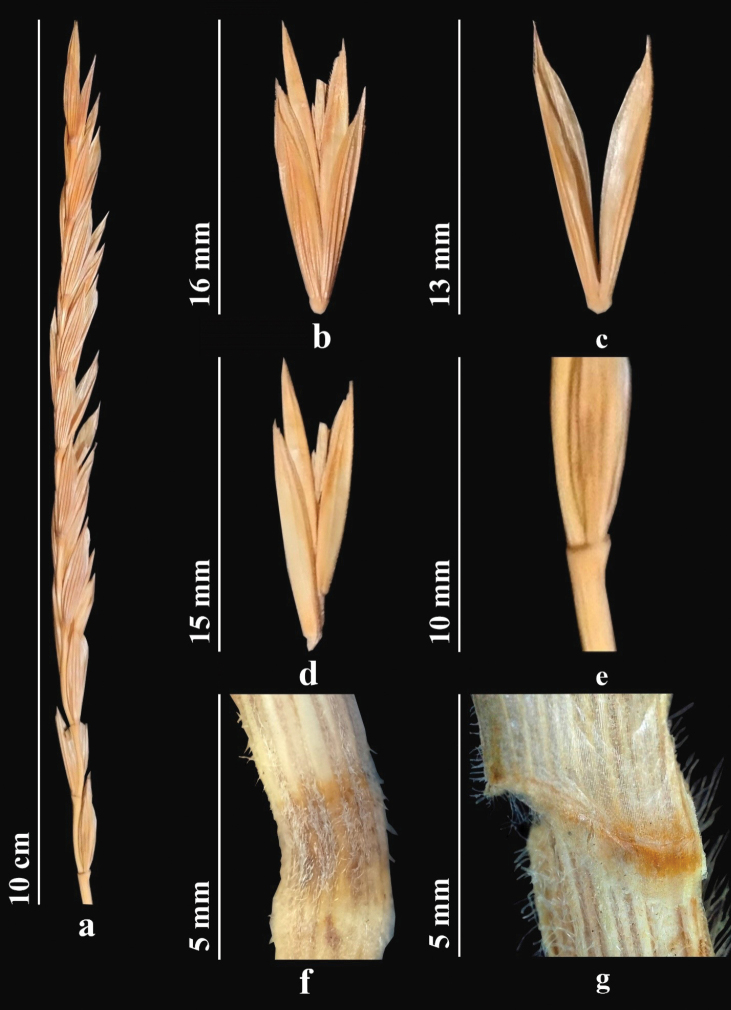
*Elymuslachnophyllus***a** spike **b** spikelet **c** glumes **d** lemmas and paleas **e** stem **f** node **g** ligule (https://doi.org/10.13140/RG.2.2.18332.30088).

Stems 60–120 cm, shortly hairy. Leaves 7–12 mm wide, flat, and short-hairy on both surfaces. Sheaths hairy. Ligule short. Spikes with closely spaced spikelets, often one-sided. Spikelets usually with 5–8 florets. Internodes of spike rough along two lateral ridges. Glumes lanceolate-oblong, intervals between veins wider than veins; apex abruptly acute. Awn of lemma straight, to 6 mm. Palea linear. Anthers 2–3 mm (Fig. 5).

##### Phenology.

Flowering and fruiting: June-August.

##### Habitat.

On rocky slopes, within *Juniperus* forests, in middle mountain belt, 2200–2600 m.

##### General distribution.

Middle Asia (Pamir-Alay: Hissar, Darvaz, Turkestan Ranges): Tajikistan, Uzbekistan.

##### Distribution in Uzbekistan.

Kashkadarya region (Fig. 3B). I-6 **Western Hissar district.** I-6-a **Kashkadarya region.** HISSAR RANGE (Western Pamir-Alay. Kyzyl-Darya River Basin. Karam-Kul tract. Eastern slope (40°) in the upper reaches of the Karam-Kul River, 01.09.1941, *Koshernikova 652* [TASH046021!]).

##### Note.

*Elymuslachnophyllus* is given here as a new record to the flora of Uzbekistan.

#### ﻿4. Elymus longe-aristatus

Taxon classificationPlantaePoalesPoaceae

(Boiss.) Tzvelev in Novosti Sist. Vyssh. Rast. 9: 62 (1972)

2FB5F19A-7CA7-5375-8674-A35383ADCFE4

≡ Agropyronlongearistatum (Boiss.) Boiss. in Fl. Orient. 5: 660 (1884). ≡ Anthosachnelongearistata (Boiss.) Nevski in Trudy Sredne-Aziatsk. Gosud. Univ., Ser. 8b, Bot. 17: 64 (1934). ≡ Brachypodiumlonge-aristatum Boiss. in Diagn. Pl. Or., ser. 1, 7: 127 (1846). ≡ Roegnerialongearistata (Boiss.) Drobow in Fl. Uzbekistan. 1: 280 (1941).
= Agropyronflexuosissimum Nevski in Izv. Bot. Sada Akad. Nauk S.S.S.R. 30: 510 (1931 publ. 1932). Type. Tajikistan. Karategin, Galagan Glacier, 10,000 ft, 07 July 1896, *V.I. Lipsky 2497* (holotype LE, isotype: LE). ≡ Elymuslongearistatussubsp.flexuosissimus (Nevski) Tzvelev in Novosti Sist. Vyssh. Rast. 10: 26 (1973).
= Agropyronlongiaristatumvar.aitchisonii Boiss., Fl. Orient. 5: 660 (1884). Type. AFGHANISTAN. Hab. ad Sergal et Sikaram vallis Kurum Affghaniae 10000’–14000’. Date unknown, *Aitch. 962*.
= Brachypodiumtataricum Munro ex Aitch. in J. Linn. Soc., Bot. 18: 109 (1880), nom. nud.
= Agropyroncanaliculatum Nevski in Izv. Bot. Sada Akad. Nauk S.S.S.R. 30: 509 (1932). Type. Tajikistan. Darvaz, Peter I range, southern slope. Vereshkay Glacier, 11,000 ft, 29 July 1899, *V.I. Lipsky 2500.* ≡ Elymuslongearistatussubsp.canaliculatus (Nevski) Tzvelev in Novosti Sist. Vyssh. Rast. 9: 62 (1972). ≡ Elymuscanaliculatus (Nevski) Tzvelev in Trudy Bot. Inst. Komarova Akad. Nauk SSSR, Rast. Tsentral. Azii 4: 220 (1968).
= Roegnerianevskiana E.Nikit. ex Zakirov in Trudy Uzbeks. Gosud. Univ. Alishera Navoi. N.S., Biol. 89: 19 (1958), nom. nud. Type. Uzbekistan. Upper Zeravshan: Saridervaza, 3500 m, 14 September 1945, Zakirov.

##### Lectotype

**(designated by Tzvelev 1972: 62).** Iran • Hab. in m. Totschal pr. Teheran, 23 VIII 1843, Pl. Pers. Bor. n°569, Th.Kotschy.

##### Description.

**Figure 6.**
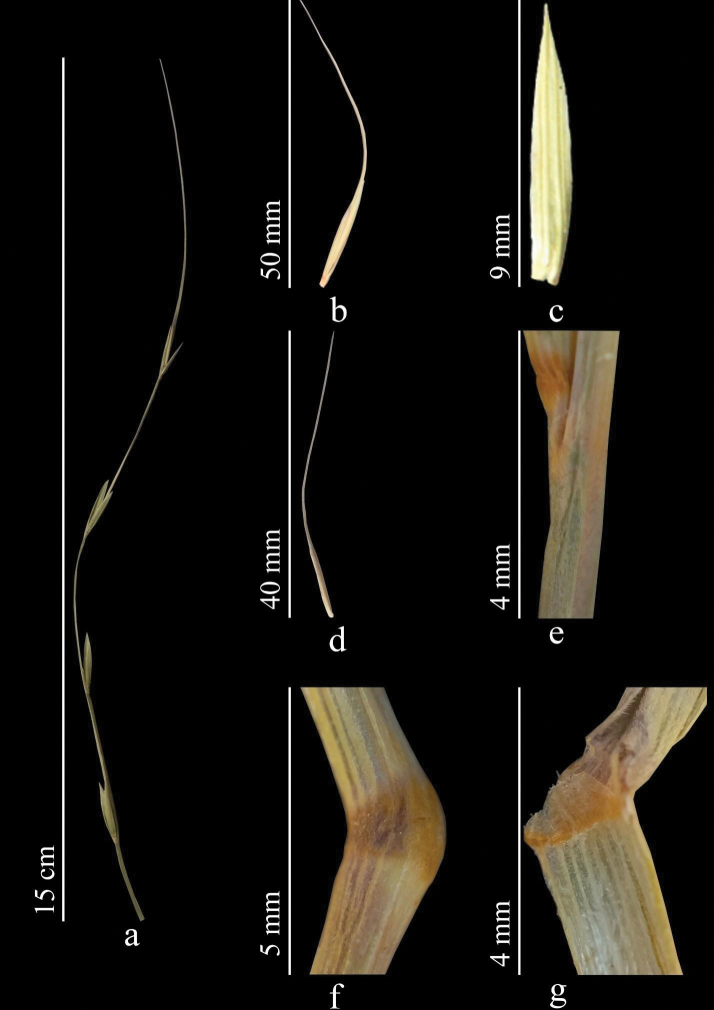
*Elymuslongearistatus***a** spike **b** spikelet **c** glumes **d** lemmas and paleas **e** stem **f** node **g** ligule. (https://doi.org/10.13140/RG.2.2.29237.49127)

Stems 30–70 cm, glabrous and smooth. Leaves folded lengthwise, glabrous, adaxially rough, abaxially smooth or occasionally hairy. Sheaths glabrous, smooth. Ligule short. Spikes drooping, with 3–7 spikelets. Spikelets solitary along axis of spike. Internodes of spikelet noticeably elongate. Glumes linear-lanceolate, apex acute or with awn to 5 (7) mm. Awn of lemma bent to side, 35–60 (70) mm. Palea linear, usually slightly shorter than lemma, ciliate along ridges. Anthers 4–6 (7) mm (Fig. 6).

##### Phenology.

Flowering and fruiting: July-August.

##### Habitat.

Middle, subalpine, and alpine belts of mountains; petrophytic communities (rocky areas, landslides, gravelly landslides, rocky-gravelly landslides, cliffs), 1200–2900 m.

##### General distribution.

Iran, Iraq, Afghanistan, Pakistan, Western Himalayas, Tibet, Nepal, Middle Asia (Hisor, Peter I, Darvoz, Kuhitang mountain ranges): Tajikistan, Turkmenistan, Uzbekistan.

##### Distribution in Uzbekistan.

Kashkadarya and Surkhandarya regions (Fig. 3D). I-6 **Western Hissar district.** I-6-a **Kashkadarya region.** HISSAR RANGE (Hazret-Sultan Mountain. Rocky slope of the Ak-su River valley. Altitude 2700 m, 17.07.1933, *Gordienko, Chilikina 214* [MW0808634]; Western Pamir-Alay. Upper reaches of the Yakkabag-Darya River. Around the village of Tash-Kurgan. Mountain Maskara. Limestone scree, 14.07.1936, *Butkov Bochantsev 937* [TASH040995!]); I-6-c **Baysun region.** HISSAR RANGE (Ketmen-Chapty Mountain, rocky slope, near the Gasa pass, 2800 m, 20.07.1935, *Gordienko 22* [MW0808631]); I-6-d **Kuhitang region.** KUHITANG RANGE (Montes meridionales: Sogdiano-transoxani: In detritu calcareo submobili in montibus Kuhitang supra p. Kizyl-alma, 28.06.1927, *Vvedensky, Popov* [TASH046135!]; Pamir-Alay Mountains, Kempir-Tyube, 15.07.1935, *Pryanishnikov 42* [MW0808633]; Kugitang-Tau. Machaily (Machaily-say) juniper forest, 2200 m, 11.05.1985, *Khasanov* s.n. [TASH126638!, TASH126639!].

#### ﻿5. Elymus macrochaetus

Taxon classificationPlantaePoalesPoaceae

(Nevski) Tzvelev in Novosti Sist. Vyssh. Rast. 9: 61 (1972)

40B356A5-AF55-5420-80B0-6F82C78E428B

≡ Agropyronmacrochaetum (Nevski) Bondarenko in Opred. Rast. Sred. Azii 1: 170 (1968). ≡ Roegneriamacrochaeta Nevski in V.L.Komarov (ed.), Fl. URSS 2: 612 (1934). ≡ Semeiostachysmacrochaeta (Nevski) Drobow in Fl. Uzbekistan. 1: 281 (1941).

##### Type.

Tajikistan • Tadshikistania orientalis. Systema fl. Jach-su; ad fl. Obi-Daschtako superiorem prope pagum Schugnau (in montibus Chasretischa), 27 September 1932, *N. Goncharov, G. Grigorjev, N.V. Nikitin 985* [K000674857].

##### Description.

**Figure 7.**
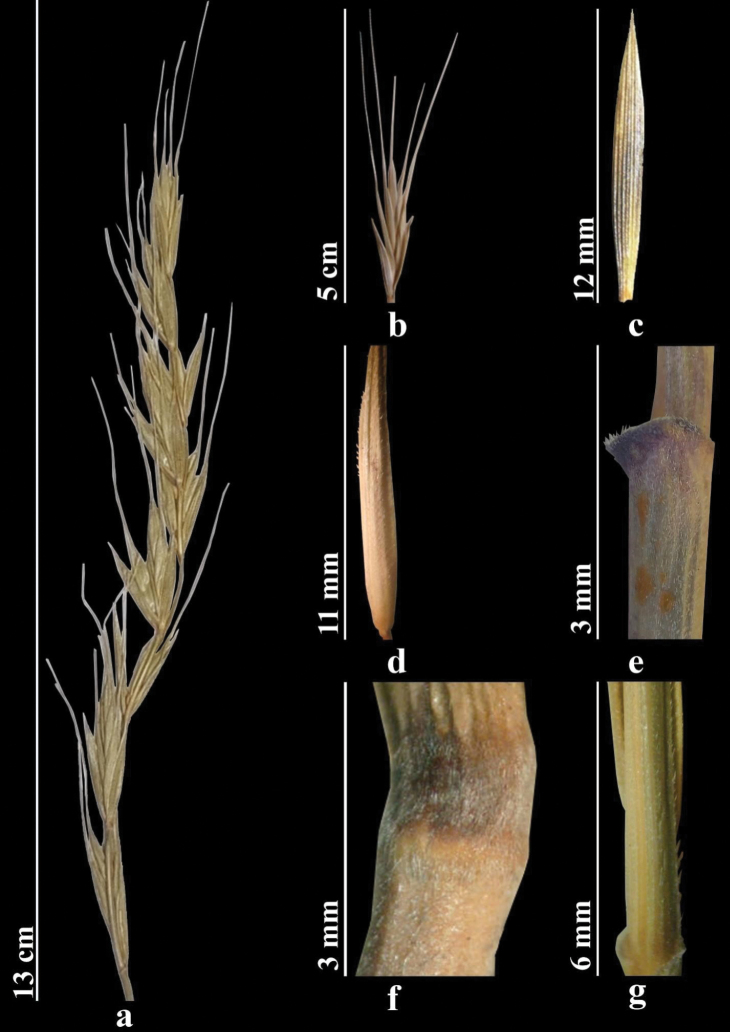
*Elymusmacrochaetus***a** spike **b** spikelet **c** glumes **d** lemmas and paleas **e** stem **f** node **g** ligule (https://doi.org/10.13140/RG.2.2.32592.93445).

Stems numerous, 80–120 cm, densely short hairy below nodes. Leaves 3–9 mm wide, adaxial surface densely hairy. Sheaths rough. Ligule truncate, to 1 mm. Spikes erect, rarely slightly drooping. Spikelets solitary along axis of spikes. Internodes of spike axis rough along two lateral ridges. Glumes 10–20 mm, almost equal, narrowly lanceolate, apex narrowly acute, with awn to 3 mm. Lemma abaxially with spines or hairs across entire surface, with awn 15–30 mm, awn straight or often slightly curved. Palea lanceolate, elongate, margins short ciliate. Anthers 3–4.5 mm (Fig. 7).

##### Phenology.

Flowering and fruiting: July-August.

##### Habitat.

Among shrubs, in sparse forests, in open forest clearings, gravel beds and rocky slopes in middle and upper mountain belts, 800–3400 m.

##### General distribution.

Middle Asia (Tian Shan (indicated for the Talas Alatau), Pamir-Alay: Hissar, Darvaz, and Western Pamir ranges): Kyrgyzstan, Tajikistan, and Uzbekistan (Alieva et al. 2024b).

##### Distribution in Uzbekistan.

Kashkadarya and Surkhandarya regions (Fig. 3E). I-6 **Western Hissar district.** I-6-a **Kashkadarya region.** HISSAR RANGE (South-western Hissar, Hissar State Nature Reserve, Tankhozdarya branch, Osmantalash ridge, 02.07.2021, *Aromov.* Southwestern Hissar, Hissar State Reserve, Gilon department, Novshur say, 14.07.2022, *Aromov*; Kyzylsuv department, big Khursanddara say, 30.07.2022, *Aromov* s.n. [Herbarium of the Hissar State Nature Reserve.]); Southwestern Hissar, Hissar State Reserve, Tankhozdarya department, Kuralai say, 30.07.2022, *Aromov* s.n. [Herbarium of the Hissar State Nature Reserve.]). I-6-c **Baysun region.** (Mountains of Khodja-gurgur ata. Basin of the Khodja-ipak say River, Kyzyl-su, 29.07.1934, *Penkaovich 148* [TASH046154!]. I-7 **Hissar-Darvaz district.** I-7-a **Sangardak-Tupalang region.** HISSAR RANGE (Sary-Asiya district. Descent from Dzhaukoz pass, 04.08.1931, *Merkulevich* s.n. [TASH046155!, TASH046156!]; Pamir-Alay. 3 km east of the village Khovat, altitude 2200 m, at the upper boundary of the deciduous forest. Description 51., 02.08.1941, *Gromakov 634* [TASH046157!]).

#### ﻿6. Elymus nevskii

Taxon classificationPlantaePoalesPoaceae

Tzvelev in Spisok Rast. Gerb. Fl. S.S.S.R. Bot. Inst. Vsesojuzn. Akad. Nauk 18: 29 (1970)

F9AA953C-45BE-5744-91AE-106524623776

= Agropyrondentatum Hook.f. in Fl. Brit. India 7: 370 (1896). Type. India. Kashmir, alt. 9–12,000 ft., Jacquemont, Thomson.
= Agropyronugamicum Drobow in A.I.Vvedensky & al., Key Fl. Tashkent 1: 41 (1923). Type. Uzbekistan. Western Tian Shan. “Distr. Tashkent, near the Ugam River,” 1921, n° 1313, Uranov. ≡ Semeiostachysugamica (Drobow) Drobow in Fl. Uzbekistan. 1: 284 (1941). ≡ Roegneriaugamica (Drobow) Nevski in Trudy Sredne-Aziatsk. Gosud. Univ., Ser. 8b, Bot. 17: 69 (1934).

##### Type.

Tajikistan • Leninabad region, southern slope of the Zeravshan range, in the basin of the Yagnob River, between the tributaries Golkrod and Yamansu, on a subalpine meadow, at an altitude of 2900 m. 18 VII 1935, *V. Nikitin* [K003372662].

##### Description.

**Figure 8.**
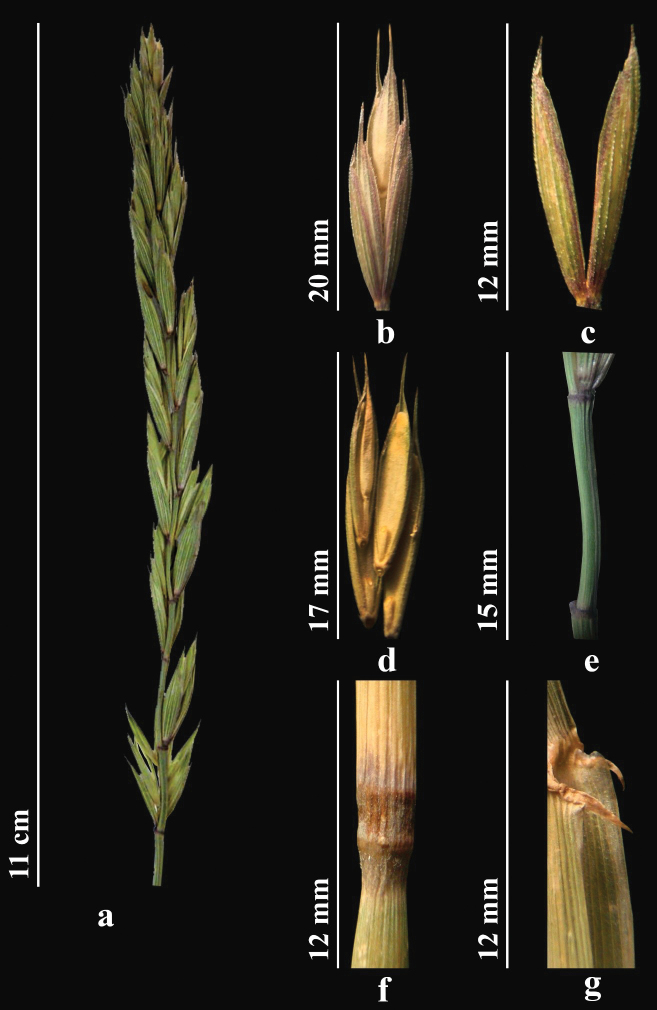
*Elymusnevskii.* a, spike **b** spikelet **c** glumes **d** lemmas and paleas **e** stem **f** node **g** ligule (https://doi.org/10.13140/RG.2.2.22526.60481).

Stems 50–120 cm, glabrous, smooth. Leaves 7–11 mm wide, flat, abaxially smooth and glabrous, adaxially rough with scattered hairs. Lowest part of sheath hairy. Ligule short. Spikes straight, 7–13 cm. Spikelets 20–30 mm, green or purple. Internodes of spike rough only along two lateral ribs. Glumes broadly lanceolate, margins membranous, quickly acuminate to bristle-like apex to 2 mm. Lemma abaxially nearly or entirely densely spiny or hairy, with awn to 7 mm. Palea linear, nearly equal to lemma. Anthers 1.8–3 mm (Fig. 8). *2n = 28* (Tzvelev and Probatova 2019).

##### Phenology.

Flowering and fruiting: June-August.

##### Habitat.

Middle, subalpine, and alpine mountain belts, among shrubs (polydominant, mesophytic deciduous), meadows (floodplain, mesophytic), petrophytic communities (rocky-gravelly scree, cliffs), and gravel, 800–3200 m.

##### General distribution.

China, Pakistan, Russia, western Himalayas, Middle Asia (Tarbagatai, Junggar Alatau, Tian Shan, Aral-Caspian Lowlands, Pre-Balkhash Deserts, Pamir-Alay: Gissar, Darvaz, and Pamir ranges): Kazakhstan, Kyrgyzstan, Tajikistan, Turkmenistan, Uzbekistan.

##### Distribution in Uzbekistan.

Jizzakh, Kashkadarya, Namangan, Surkhandarya, and Tashkent regions (Fig. 3F). I-1 **Western Tian Shan district.** I-1-a **Ugam-Pskem region.** UGAM RANGE (Syr-Darya Region, Tashkent District. Valley of the Ugam River. 06.1921, *Maksimov* s.n. [MW0808955]; Valley of the Pskem River. Babajan Sai (left tributary of the Pskem River). On turf-covered slopes, 26.07.1941, *Momotov 84* [TASH055307!]; Syr Darya Region, Tashkent District. Valley of the Ugam River, *Maksimov* s.n. [TASH055308!]; Bostandyk, on rocky slopes along the ridge of the Ugam Range in the upper reaches of the Navali-Sai gorge, at 2800–3000 m, 31.07.1953, *Pavlov 467* [MW0808951, MW0808959]; Oygaing, 03.08.2023, *Turdiev, Aliyeva O_16_A* [TASH054576!]); PSKEM RANGE (Bostandyk, on rocky scree beneath a large snow patch at the head of Aksar-Sai, 24.07.1949, *Pavlov 158* [MW0808948], 28.07.1949, *Pavlov 244* [MW0808949, MW0808950]; Upper reaches of the Oygaining River. Gentle slopes of the right bank of Tunduk-Sai, 20.08.1956, *Tsukervanik 1572* [TASH055292!], *Granitov 1567* [TASH055296!]). I-1-b **Western Chatkal region**. CHATKAL RANGE (Chatkal Mountain-Forest Reserve. Maidan-Tal section, Tashkent-Say, southern slope at an altitude of 1500–1700 m, 10.10., *Savich* s.n. [TASH046203!, TASH046204!]; Vicinity of the Chimgan Botanical Station. Gorge of the Chimganka River, on the stream bank near the waterfall, 26.08.1928, *Gomolitsky 477* [TASH055301!]; Myn-Dzhilke tract. Upper reaches of the Nurek-Ata River. Southern gravelly slopes (does not fit the description, as there is no awn on lemma), 17.07.1936, *Korotkova, Titov 1430* [TASH047501!], *1446* [TASH047502!]; Basin of the Angren River. Rocky slope below the Davan-Sai Pass, 15.08.1937, *Zakirov* s.n. [SAMDU]; Basin of the Chatkal River. Mazar River Valley. Eastern slope of the left bank of the Mazar River, belt of creeping form juniper, 08.08.1938, *Pyataeva, Momotov 604* [TASH055304!]; Tashkent Alatau Mountains. Basin of the Bash-Kyzyl-Say River. Menora-Say, 02.08.1953, *Butkov, Tsukervanik 1203* [TASH046343!]; New section of Reserve, Chatkal Mountains, 70 km E of Tashkent, *314209* [USDA_NPGS3417113], *314200* [USDA_NPGS3417106], *314211* [USDA_NPGS3417103]); I-1-c **Arashan region**. CHATKAL RANGE (Angren Expedition. Right bank of the Angren River; warm springs of Arasan, granite, 14.08.1924, *Korovin 2736* [TASH055326], 16.08.1924, *Korovin 2737* [TASH055327]; Basin of the Angren River. Upper reaches of the Angren River, above the warm springs of Arashan, along the shore of the lake, at an altitude of 1750 m. 28.07.1938, *Pyataeva, Momotov 347* [TASH055329!, TASH055330!]; Angren Plateau. Southern slopes. Near Arasan Lake. Northeastern slope. Alpine vegetation belt, 19.08.1939, *Kudryashev 1237* [TASH055331!]; Arashan Lake, on the slopes, 24.07.1940, *Korotkova 231* [TASH047493!]); I-1-d **Kurama region.** CHATKAL RANGE (Basin of the Angren River. Upper reaches of the Angren River, above the warm springs of Arashan, sand deposits on the southeast slope of the Babai-Tagh ridge, site near the water, 28.07.1938, *Pyataeva, Momotov 316* [TASH046200!]; Tashkent Alatau. Southern gravelly slope of Mountain Kyzyl-nura, altitude 3200 m, 11.09.1939, *Gomolitsky 77* [TASH055309!]; Basin of the Angren River. In the upper reaches of the Chet-su, on gravelly slopes, 27.07.1940, *Korotkova 315* [TASH055335!]; Valley of the Angren River. Sai Ayri. Tugai, 17.08.1940, *Usmanov 1187* [TASH055386!]); KURAMA RANGE (Kishvash Sai, upper reaches of Lashkerek, above the juniper zone. Rocky slope, 12.07.1939, *Zakirov* s.n. [SAMDU]; Basin of the Angren River. Dawan-sai, rocky slope below the pass, 15.08.1937, *Zakirov* s.n. [TASH055328!]; Northern slopes of the upper reaches of Nizbash-sai. Eastern slope with subalpine vegetation, 06.08.1939, *Kudryashev 1083* [TASH055336!, TASH055337!]; Northern slope of Say Saracha, western slope of the Juniper forest, 13.07.1940, *Usmanov 1307* [TASH055390!]; Northern slope of Say Kum-Kul. Tugay vegetation, 10.08.1940, *Usmanov 1210* [TASH055387!]; Northern slopes of the Kurama Range, Abyazsay, 4^th^ brigade of the breeding sovkhoz, h = 1950 m, 11.09.1956, *Kamalov 136* [TASH055389!], h = 1900 m, juniper zone (prostrate form), of the breeding farm, 11.09.1956, *Kamalov, Vernik, Nabiev, Tsukervanik 137* [TASH055388!], Stud farm, rocky gravelly slope, 2150 m, 17.09.1956, *Kamalov 316* [TASH055385!]). I-5 **Kuhistan district.** I-5-a **North Turkestan region.** TURKESTAN RANGE (Zaamin Forestry, Gorge of the Kul-su River, 23.07.1926, *Popov, Androsov 116* [TASH055323!], *117* [TASH055324!]; Gurlash River Gorge, on the left bank of the Gurlash River, opposite Chutka-Say, 25.07.1926, *Popov, Androsov 144* [TASH055325!]; Zaamin District. Headwaters of the Chandyir Rivers, near the pass. Absolute altitude 2700–3000 m, 14.09.1932, *Titov, Eliseeva 600* [TASH055322!]; Pamiro-Alay. Basin of Guralash River. Cone of the Mechetyly-Say River, 27.07.1934, *Zakrzhevskiy 491* [TASH055316!, TASH055319!]; Northern slopes of the Turkestan Range, headwaters of the Sanzar River. Guralash-Say Nature Reserve. At the head of Guralash, on the northern slopes of Langar-Say, 14.08.1937, *Korotkova, Vasil’kovskaya 1039* [TASH055341!], 17.08.1937, *Korotkova, Vasil’kovskaya 1073* [TASH055320!]; along the ridge of Angerly-Say, opposite Shibarly-Say. Dry subalpine meadow, 30.08.1947, *Nazarenko* s.n. [TASH055237!, TASH055238!]; 31.08.1947, *Nazarenko 634* [TASH055235!]); I-5-b **Malguzar region.** MALGUZAR RANGE (Northern slopes of the Turkestan Range. 4–5 km from the village of Besh-Kubu towards the Zaamin mountains, 22.05.1937, *Korotkova, Vasil’kovskaya 989* [TASH055338!]). I-6 **Western Hissar district.** I-6-a **Kashkadarya region.** HISSAR RANGE (Mountain Hazret-Sultan. Aksu River. Elevation 2800 m, 17.07.1933, *Gordienko, Chilinyn 277* [MW0808944]). I-7 **Hissar-Darvaz district.** I-7-a **Sangardak-Tupalang region.** HISSAR RANGE (Pamiro-Alay. Upper reaches of the Yagly-Khocha [Yangiklik] River, elevation 3200 m. Description N82, 14.08.1941, *Gromakov 722* [TASH055318!]; Basin of the Tupolang River. Upper reaches of the Khovar River. Eastern slope in the alpine zone, 09.09.1947, *Pyatayeva 603* [TASH040959!]; Above Dara-Say at the pass. Subalpine and alpine zones, 17.07.1948, *Pyatayeva 1084*! [TASH055317!]).

#### ﻿7. Elymus praeruptus

Taxon classificationPlantaePoalesPoaceae

Tzvelev in Novosti Sist. Vyssh. Rast. 9: 61 (1972)

5C1787B9-F732-59C2-9E15-CF430A71C48F

≡ Agropyroninterruptum Nevski in Izv. Bot. Sada Akad. Nauk S.S.S.R. 30: 632 (1931 publ. 1932). ≡ Roegneriainterrupta (Nevski) Nevski in Trudy Sredne-Aziatsk. Gosud. Univ., Ser. 8b, Bot. 17: 68 (1934). ≡ Semeiostachysinterrupta (Nevski) Drobow in Fl. Uzbekistan. 1: 282 (1941).

##### Type.

Uzbekistan • Samarkand region and surroundings, Zimarl on Djidjik-Rute, meadow by the stream, 12 VII 1913, *B. Fedtschenko 183* (holotype: LE).

##### Description.

**Figure 9.**
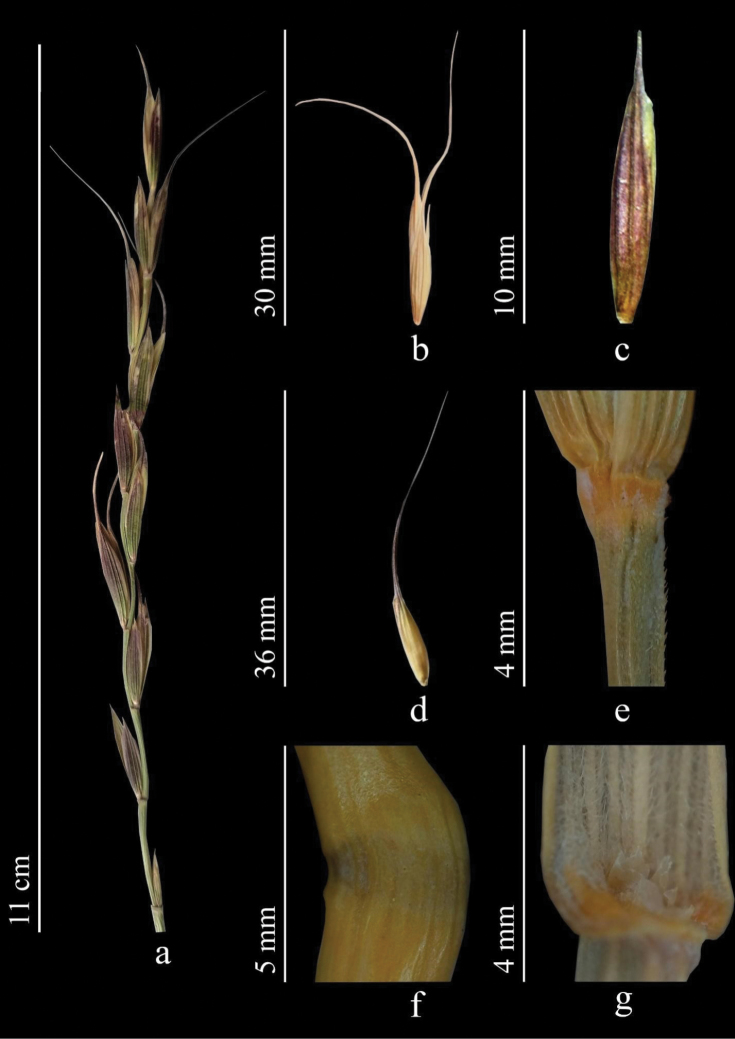
*Elymuspraeruptus***a** spike **b** spikelet **c** glumes **d** lemmas and paleas **e** stem **f** node **g** ligule (https://doi.org/10.13140/RG.2.2.11621.41442).

Stems 30–150 cm. Leaves 1.2–4 mm wide, often longitudinally folded, grayish green, adaxially with fairly prominent ribs, densely short hairy. Sheaths glabrous or hairy. Ligule to 1.2 mm. Spikes erect. Spikelets solitary along axis of spike. Lower internodes of rather loose spikes smooth or with scattered short spines along ribs. Glumes broadly lanceolate, margins membranous, apex rapidly acuminate. Lemma with scattered spines, often with bluish bloom; awn more or less bent to side, 20–30 mm. Palea linear, nearly equal to lemma. Anthers 3–4.5 mm (Fig. 9).

##### Phenology.

Flowering and fruiting: June-July.

##### Habitat.

On rocky slopes, cliffs, meadows, and gravelly areas, in middle and upper mountain belts, 1500–3100 m.

##### General distribution.

Middle Asia (Western Tian Shan, Pamir-Alay: Alay, Hissar, Darvaz mountain ranges): Kyrgyzstan, Tajikistan, Uzbekistan.

##### Distribution in Uzbekistan.

Jizzakh, Kashkadarya, Namangan, Surkhandarya, Tashkent, and Fergana regions (Fig. 3G). I-1 **Western Tian Shan district.** I-1-a **Ugam-Pskem region.** PSKEM RANGE (Upper reaches of the Oygaing River. Slopes of the right bank of Tundyk-say, 21.08.1956, *Granitov 1615* [TASH040969!]; Oygaing River valley, upper reaches of the Tunduksay gorge. Altitude 2269 m, 10.08.2019, *Tojibayev, Juramurodov 1008103* [TASH060161!]; Upper reaches of the Barkraksay gorge. Altitude 2540 m, 11.08.2019, *Tojibayev, Juramurodov 1108167* [TASH060160!], [TASH060159!]); I-1-b **Western Chatkal region.** CHATKAL RANGE (Mik-dzhilke tract. Upper reaches of the Nurek-ata River. Gravelly slopes, 21.07.1936, *Korotkova, Titov 1602* [TASH040943!]); I-1-c **Arashan region.** CHATKAL RANGE (Angren Expedition. Syr-Darya region, Tashkent district. Pass Arasan, altitude 2500 m, and the slopes leading to it, 17.08.1924, *Sovetkina 192* [TASH040949!, TASH040952!]); I-1-d **Kurama region.** CHATKAL RANGE (Southern slopes. Pass from Angren to Parkent. Substrate: rocky-gravelly, with pebbles, 22.07.1939, *Kudryashev 878* [TASH040937!, TASH040954!]; Basin of the Angren River. Pass between Bashkutan-say and Aksu-say. On a fine-soil and gravelly slope, 01.08.1954, *Butkov, Mailun 577* [TASH040945!, TASH040955!]). I-3 **Fergana-Alay district.** 1-3-b **Eastern-Alay region.** ALAY RANGE (Skobelievsky District. Northern slope of the Alay Range. Basin of the Shakhimardan River. Upper reaches of the Shivali River. Rocky slope with juniper tree, 25.07.1915, *Drobow 271* [TASH045196!]). I-5 **Kuhistan district.** I-5-a **North Turkestan region.** TURKESTAN RANGE (Zaamin Forestry. Gorge of the Kul-su River, 26.07.1923, *Popov, Androsov 99* [TASH045189!]; *118* [TASH045190!]; Zaamin Forestry. Gorge of the Gurulash River, 26.07.1923, *Popov, Androsov* s.n. [TASH045187!]; 26.07.1926, *Popov, Androsov* s.n. [TASH045188!]; Gallya-Aral District. Mountain Chumkar-Tau. Ridge between the passes Urmitan and Guralash. (Absolute height 3000–3100 m), 16.08.1932, *Titov Eliseeva 295* [TASH045194!, TASH045195!]; Northern slopes of the Turkestan Range, upper reaches of the Sanzar River. Guralash-Sai Nature Reserve. On the southeast slope, burned areas, 09.08.1937, *Korotkova, Vasilykovskaya 954/а* [TASH045191!]; At the head of Guralash. Watershed between the Langar and Kichik-Shibarly ranges, 13.08.1937, *Korotkova, Vasilykovskaya 1003* [TASH040939!]; Along the red scree on the left side of Angirly-Sai, 28.08.1937, *Korotkova, Vasilykovskaya 1187* [TASH045192!, TASH045193!]). I-6 **Western Hissar district.** I-6-a **Kashkadarya region.** HISSAR RANGE (Northern slopes of the Hissar Range. Basin of the Kashkadarya River. Gorge of the Tamshush River (tributary of Ak-su). Near the Tamshush Pass. Alpine belt. Rocks, 03.08.1937, *Kudryashev 1373* [TASH040944!, TASH040953!]). I-7 **Hissar-Darvaz district.** I-7-a **Sangardak-Tupalang region.** HISSAR RANGE (Basin of the Tupalang River. Upper reaches of the Khovat River. Right bank of the Khovat River in the alpine zone, 10.09.1947, *Pyataeva 631* [TASH040960!]).

#### ﻿8. Elymus tianschanigenus

Taxon classificationPlantaePoalesPoaceae

Czrep., Sosud. Rast. SSSR: 351 (1981). Typonym: Agropyron tianschanicum Drobow.

323D371C-69BF-5AAD-B007-5B23A9582C7B

≡ Agropyrontianschanicum Drobow in A.I.Vvedensky & al., Key Fl. Tashkent 1: 40 (1923). ≡ Roegneriatianschanica (Drobow) Nevski in Trudy Sredne-Aziatsk. Gosud. Univ., Ser. 8b, Bot. 17: 17 (1934). ≡ Semeiostachystianschanica (Drobow) Drobow in Fl. Uzbekistan. 1: 284 (1941). ≡ E.uralensissubsp.tianschanicus (Drobow) Tzvelev in Novosti Sist. Vyssh. Rast. 10: 22 (1973).

##### Lectotype

**(designated by Tzvelev 1976: 116).** Uzbekistan • Western Tian Shan. Tashkent District, Khumsan, Haudale Mountains, 14 VIII 1920, *No. 1254, M. Popov* [TASH0000162!].

##### Description.

**Figure 10.**
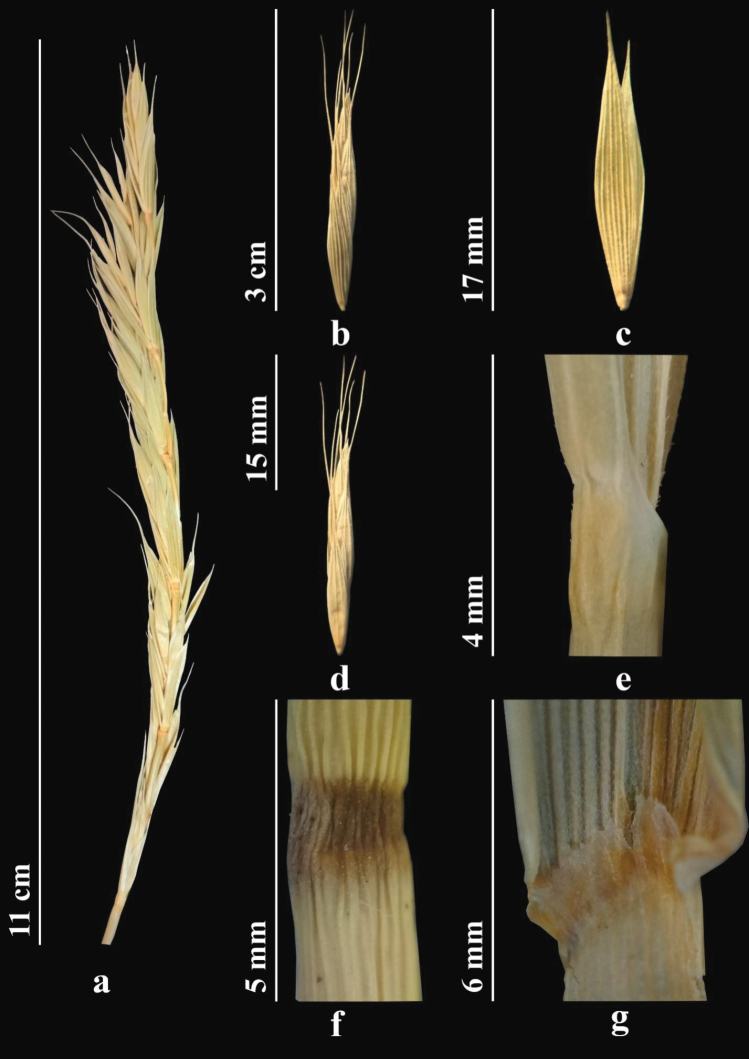
*Elymustianschanigenus***a** spike **b** spikelet **c** glumes **d** lemmas and paleas **e** stem **f** node **g** ligule (https://doi.org/10.13140/RG.2.2.31754.07360).

Stems 50–100 cm, glabrous, smooth. Leaves flat, linear, abaxially rough, glabrous or sparsely hairy. Sheath glabrous, smooth. Ligule to 1 mm. Spikes erect, rarely slightly drooping. Spikelets solitary along axis of spikes. Glumes broadly lanceolate, abruptly acute, rough. Lemma adaxially hairy on most of surface; awn straight or often slightly twisted, to 15 mm. Palea nearly equal to lemma, slightly notched, with ciliate keels. Anthers to 3 mm (Fig. 10).

##### Phenology.

Flowering and fruiting: July-August.

##### Habitat.

Among shrubs (polydominant, mesophytic deciduous), meadows (floodplain, mesophytic), petrophytic associations (rock scree, gravel scree), in lawns, pebble beds, rocky slopes, forest clearings, among shrubs; in middle and upper mountain belts, 900–3000 m.

##### General distribution.

Russia, China (Kashgar), Mongolia, Middle Asia (Tarbagatai, Dzungarian Alatau, Tian Shan, Pamir-Alay: Hissar, Darvaz, and Pamir Ranges); Kazakhstan, Kyrgyzstan, Tajikistan, Turkmenistan, Uzbekistan.

##### Distribution in Uzbekistan.

Jizzakh, Namangan, and Tashkent regions (Fig. 3J). I-1 **Western Tian Shan district.** I-1-a **Ugam-Pskem region.** UGAM RANGE (Bostandyk. On the rocks along the ridge of the Ugam Mountain range in the upper reaches of the Navali-say gorge, 2800 m, 31.07.1953, *Pavlov 466* [MW0808858]); PSKEM RANGE (Pskem River basin. Middle course of the Ikhnach-say rivers, 31.07.1941, *Momotov 171* [TASH047495!]; Tashkent region, Bostandyk district. Upper reaches of the Pskem, Tyuytash-say, 1955, *Karpeshko* s.n. [TASH044516!]; Middle part of the Pskem River valley. Western slope on the right bank of the Ispay-say, 21.07.1956, *Tsukervanik 1249* [TASH043752!]; Barkraksay, h = 2300 m, 14.08.2002, *Maltsev* s.n. [TASH060361!]); MAYDANTAL RANGE (Bostandyk district. Valley of the Oygaing River (right bank), 6–7 km above the Beshstor gorge. On a stony slope, 1700 m, 18.07.1958, *Pavlov 44* [MW0808857]; 18.08.1958, *Pavlov* 44 [MW0808856]). I-1-b **Western Chatkal region**. CHATKAL RANGE (Tian-schan occidentalis. Chatkal Mountain Forest Reserve. Maydantal section. Tashkentsay, h = 1500–1700 m. Southern slope, 10.10., *Savich* s.n. [TASH043754!]; Tashkent Alatau Mountains. Basin of the Nurekata River, Almalyk-say, 30.07.1953, *Maylun, Nabiev, Tsukervanik 1000* [TASH043726!]; I-1-d **Kurama region.** KURAMA RANGE (Upper reaches of the Angren River. Angren Plateau. Southern slopes. Upper part of Karasay. Dry slopes, 21.08.1939, *Kudryashev 1331* [TASH047503!]); CHATKAL RANGE (Tashkent Alatau. Parkent district. Mountain Takali. Rocky-gravel slope, 12.08.1953, *Butkov, Tsukervanik 1224* [TASH047494!]); I-1-e **Chorkesar region.** KURAMA RANGE (Foothills of the Fergana Range. Around the village of Gava, 09.07.1928, *Kryltsova 509* [TASH043779!]). I-5 **Kuhistan district.** I-5-a **North Turkestan region.** TURKESTAN RANGE (Pamir-Alay. Basin of the Zaamin-su River. Valley of the Tuyatashsay River, 18.07.1935, *Zakrzhevsky 963* [TASH047499!], *970* [TASH055321!]; Reserve “Guralash,” Kul-Say, 2400 m, 15.07.1940, *Kultiassov* s.n. [MW0808851]; Guralash Reserve. Tuyatash-say, 14.06.1947, *Nazarenko 253* [TASH047500!]; Kul-Say. Juniper forest. Northern slope, 2300 m, 07.09.1954, *Obongetskaya 212* [TASH047497!]; Kul-Say. Southern slope, 2300 m, 09.09.1954, *Obongetskaya 214* [TASH047498!]).

#### ﻿9. Elymus transhyrcanus

Taxon classificationPlantaePoalesPoaceae

(Nevski) Tzvelev in Novosti Sist. Vyssh. Rast. 9: 61 (1972)

048DBF6C-2310-5B37-9B6B-4A8B603D7713

≡ Agropyrontranshyrcanum (Nevski) Bondarenko in Opred. Rast. Sred. Azii 1: 173 (1968). ≡ Roegneriatranshyrcana Nevski in Trudy Sredne-Aziatsk. Gosud. Univ., Ser. 8b, Bot. 17: 70 (1934).
= Elytrigiavvedenskyi Drobow in Fl. Uzbekistan. 1: 539 (1941). Type. Uzbekistan. Chulbair Mountains. Valley of the Obi-Dara River near the village of Sina. 31 V 1929, *Vvedensky, 184*.
= Roegnerialeptoura Nevski in V.L.Komarov (ed.), Fl. URSS 2: 623 (1934). Type. Turkmenistan. Mountains of Chapan-Dag (Kopet-Dag). Collectorunknown (holotype LE). ≡ Semeiostachysleptoura (Nevski) Drobow in Fl. Uzbekistan. 1: 285 (1941).

##### Type.

Turkmenistan • Ashgabat District, rocky areas at an elevation of 1000 m, Mountain Chapandag, 25 VIII 1931, No. 725, *A. Borisova* (isotype LE).

##### Description.

**Figure 11.**
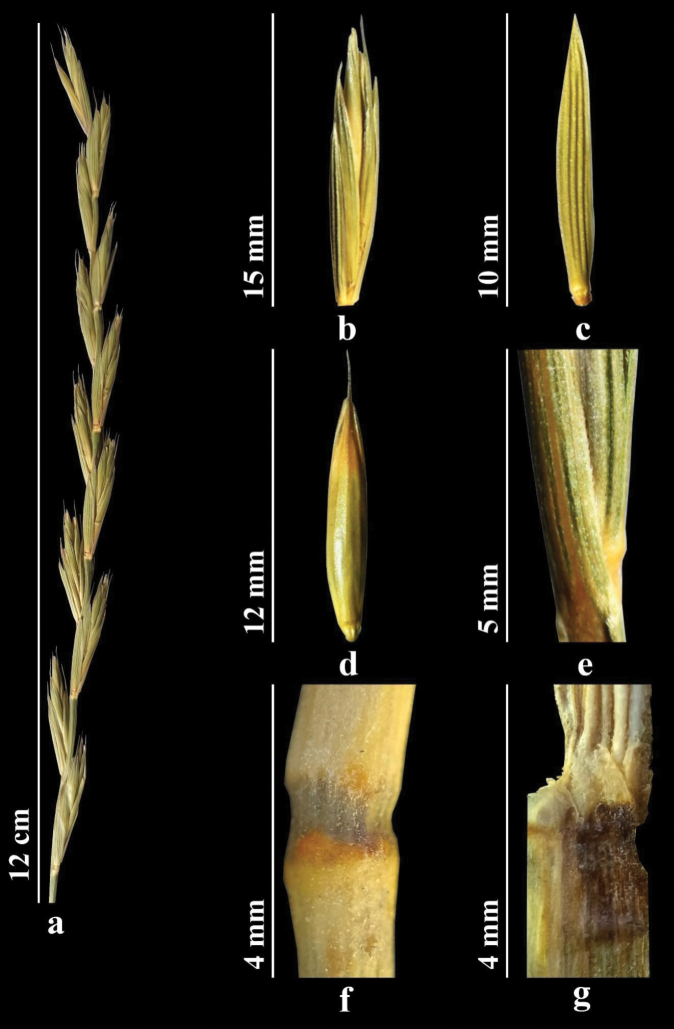
*Elymustranshyrcanus***a** spike **b** spikelet **c** glumes **d** lemmas and paleas **e** stem **f** node **g** ligule (https://doi.org/10.13140/RG.2.2.14976.85763).

Stems 80–95 cm, glabrous, smooth. Leaves 1.4–4 mm wide, grayish green, flat, margin rolled, adaxially rigid, abaxially slightly rough or glabrous. Sheath glabrous, smooth. Ligule to 1 mm. Spikes upright, rarely slightly drooping. Spikelets solitary along axis of spikes. Internodes of spike short hairy. Glumes almost equal, narrowly lanceolate, acuminate, with awn to 3 mm. Lemma lanceolate or narrowly lanceolate, slightly rough or glabrous, apex acute, with awn to 2 mm. Palea narrowly lanceolate, margins short ciliate. Anthers 3.5–4.5 mm (Fig. 11). *2n = 56* (Tzvelev 1976).

##### Phenology.

Flowering and fruiting: June-August.

##### Habitat.

On rocky slopes, cliffs, and pebbles in middle and upper mountain belts, 1700–2400 m.

##### General distribution.

Eastern and southern Transcaucasia, Iran, Turkey, Middle Asia (Tian Shan, Pamir-Alay: Hissar, Darvaz Ranges): Kazakhstan, Tajikistan, Turkmenistan, Uzbekistan.

##### Distribution in Uzbekistan.

Jizzakh, Kashkadarya, Surkhandarya, and Tashkent regions (Fig. 3H). I-1 **Western Tian Shan district.** I-1-b **Western Chatkal region**. CHATKAL RANGE (Surroundings of the Chimgan Botanical Station. Big Chimgan, 07.1929, *Gomolitsky* s.n. [TASH047581!]; Kashka-Su River Gorge, 26.07.1936, *Korotkova, Titov 1702* [TASH047578!]; Ridge between the upper Bashkizylsay and Chouli. Northern slopes, 29.07.1936, *Korotkova, Titov 1847* [TASH047579!]; Basin of the Chatkal River. Valley of the Akbulak River. South-Western slope of the first watershed ridge of the upper Akbulak River. Shrub and tall grass vegetation, 03.09.1938, *Pyatayeva, Momotov 1564* [TASH047570!]; Reserve, Chatkal Mountains, 70 km E of Tashkent *314199* [USDA_NPGS3436603], *314202* [USDA_NPGS3436605]; New section of Reserve, Chatkal Mountains, 70 km E of Tashkent *314206* [USDA_NPGS3436628], *314208* [USDA_NPGS3436624]); I-1-d **Kurama region.** KURAMA RANGE (Northern slopes. Near the Shaugaz Pass. Northern slope. Soil: fine-grained, brown, mountain-forest, 10.07.1939, *Kudryashev 553* [TASH046184!, TASH046185!]). I-5 **Kuhistan district.** I-5-a **North Turkestan region.** TURKESTAN RANGE (5–6 km south of the village of Tenga-Tapty, 19.08.1937, *Demurina 1167* [TASH047582!]; Northern slopes of the Turkestan Range, headwaters of the Sanzar River. Guralash-Say Nature Reserve. On the northeastern slope of Angyrly-Say, 26.08.1937, *Korotkova, Vasil’kovskaya 1143* [TASH047576!]). I-6 **Western Hissar district.** I-6-a **Kashkadarya region.** HISSAR RANGE (Western Pamiro-Alay. Upper reaches of the Yakabagh-Darya River. Surroundings of the village of Tash-Kurgan. Kapyr-Say locality, 04.07.1936, *Bochantsev, Butkov 625* [TASH047574!, TASH047575!]; Northern slopes of the Gissar Range. Basin of the Kashka-Darya River. Upper part of the Tanhas River basin. Headwaters of the Tanhas River, 01.08.1937, *Kudryashev1306* [TASH047572!, TASH047573!, TASH047577!]; 02.08.1937, *Kudryashev 1339* [TASH055428!, TASH055429!, TASH055430!]); I-6-c **Baysun region.** HISSAR RANGE (Chulbair Mountains. Valley of the Obi-Dara River above the village of Sina, 31.05.1929, *Vvedensky 184* [TASH0000179!, TASH0000180!]). I-7 **Hissar-Darvaz district.** I-7-a **Sangardak-Tupalang region.** HISSAR RANGE (Pamiro-Alay. Gissar Range, Delli-Say, above the mouth of the Khucha River, elevation 2300 m. Description No. 87, 16.08.1941, *Gromakov 729* [TASH047583!]; Basin of the Tupolang River. Valley of the Khovat River. Stream on the right bank of the Khovat River, at the confluence of the Artushgar stream, 06.09.1947, *Pyatayeva 483* [TASH047583!]; Headwaters of the Shatrut River, upstream along the Chilik-Su River to the glaciers, and the surroundings of Chirmak-Zor, 28.06.1948, *Pyatayeva 817* [TASH047584!].

#### ﻿10. Elymus tschimganicus

Taxon classificationPlantaePoalesPoaceae

(Drobow) Tzvelev in Trudy Bot. Inst. Komarova Akad. Nauk SSSR, Rast. Tsentral. Azii 4: 221 (sub « E. czimganicus») (1968), orth. var.

CD7AB472-0A71-5D10-9B90-2C4E85AAB30D

≡ Agropyronczimganicum Drobow in M.G.Popov (ed.), Key Pl. Envir. Tashkent: 40 (1923). ≡ Roegneriaczimganica (Drobow) Nevski in V.L.Komarov (ed.), Fl. URSS 2: 604 (1934).

##### Type.

(lectotype designated by Drobow 1925: 41). Uzbekistan • Prov. Syr-darja. Distr. Taschkent. In montibus ca. urb. Taschkent. *Popov*, 1921, no. 1266 et 1270 [TASH000146 and TASH000147]).

##### Description.

**Figure 12.**
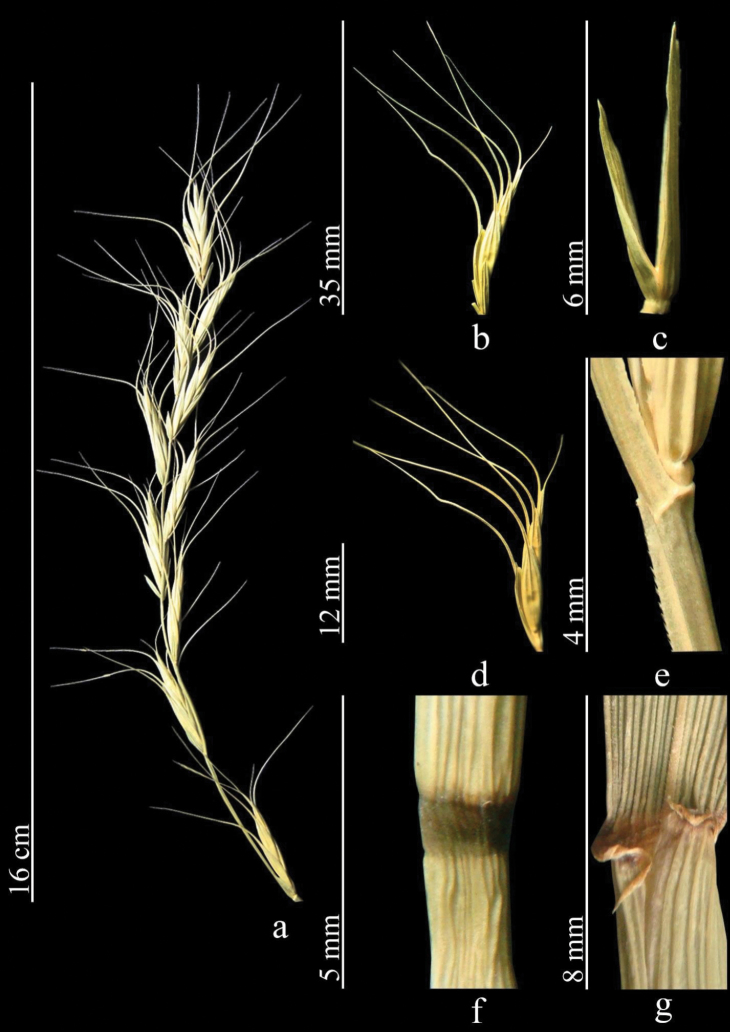
*Elymustschimganicus***a** spike **b** spikelet **c** glumes **d** lemmas and paleas **e** stem **f** node **g** ligule (https://doi.org/10.13140/RG.2.2.25043.18724)

Stems 30–70 cm, glabrous, smooth. Leaves 1.2–4 mm wide, flat or longitudinally folded, bluish green, glabrous or slightly hairy adaxially. Sheaths glabrous, smooth, lowest sheaths hairy. Ligule nearly inconspicuous. Spikes drooping. Spikelets solitary along axis of spike. Internodes of spikelet more or less short. Glumes awnless, but sometimes with mucro to 0.8 mm. Awn of lemma geniculate, bent outward, to 30 mm. Palea linear, nearly equal to lemma, apex slightly notched or obtuse. Anthers 2–2.5 mm (Fig. 12). *2n = 28* (Tzvelev 1976).

##### Phenology.

Flowering and fruiting in June-July.

##### Habitat.

In foothills, middle-subalpine and alpine mountain belts, in petrophytic communities (rocky screes, gravelly screes, rocky-gravelly screes, cliffs), meadows (floodplains, mesophytic), and gravel, 500–3400 m.

##### General distribution.

China (Kashgar), western Himalaya, Middle Asia (Tarbagatai, Dzungarian Alatau, Tian Shan, Pamir-Alay: Alay, Gissar ranges; Darvaz): Kazakhstan, Kyrgyzstan, Tajikistan, Uzbekistan.

##### Distribution in Uzbekistan.

Jizzakh, Kashkadarya, Namangan, Samarkand, Tashkent, and Fergana regions (Fig. 3I). I-1 **Western Tian Shan district.** I-1-a **Ugam-Pskem region.** UGAM RANGE (Tashkent district, Khumsan, Haudale mountains, 14.08.1920, *Popov 1266* [TASH0000146!], *1270* [TASH0000147!]); PSKEM RANGE (Bostandyk district. Upper reaches of the Barkrak-say gorge. Southern rocky slope near the GRP base, 3300 m, 08.08.1959, *Pavlov 19* [MW0808515]; Upper reaches of the Barkrak-say gorge. On the rocky southern slope near the cliffs along the right bank of the gorge near the end of the glacier, 3400 m, 11.08.1959, *Pavlov 116* [MW0808517]); I-1-c **Arashan region.** CHATKAL RANGE (Angren, surroundings of the Arаshan peak, 29.07.1938, *Zakirov 26* [SAMDU]); I-1-d **Kurama region**. KURAMA RANGE (Angren River, 1937, *Zokirov* s.n. [SAMDU]). I-3 **Fergana-Alay district.** 1-3-b **Eastern-Alay region.** ALAY RANGE (Skobelevsky district. Northern slope of the Alay Range. Basin of the Shakhimardan River. Upper reaches of the Archa-bashi River. Granite scree near vegetation, 31.07.1915, *Drobow 342* [TASH047390], *6076* [US2530891]). I-5 **Kuhistan district.** I-5-a **North Turkestan region.** TURKESTAN RANGE (Northern slopes of the Turkestan Range, upper reaches of the Sanzar River. Guralash-Say Nature Reserve. On the road, at the Guralash Pass. Altitude 2900 m, 08.09.1938, *Korotkova 278* [TASH040990, TASH040991, TASH040992]; At the summit of the watershed between Kulsay and Guralash, 14.07.1938, *Korotkova 256* [TASH041000, TASH041001]). I-6 **Western Hissar district.** I-6-a **Kashkadarya region.** HISSAR RANGE (Western Pamir-Alay. Upper reaches of the Yakkabag-Darya River. Near the village of Tash-Kurgan, 16.07.1936, *Butkov, Bochantsev 982* [TASH040994]; Outcrops of variegated rocks to the west of the village of Tash-Kurgan. Among the cliffs, 30.06.1936, *Butkov, Bochantsev 513* [TASH040997, TASH040998]); I-6-b **Tarkapchigay region.** HISSAR RANGE (Pamir-Alay. Variegated foothills to the southeast of the city of Guzar. Shale screes along the slope of Mountain Kara-San, 09.09.1935, *Lepeshkin 9* [TASH040999]).

#### ﻿11. Elymus uzbekistanicus

Taxon classificationPlantaePoalesPoaceae

Usupbaev & Alieva sp. nov.

EB34E063-77E5-58D8-A9F1-5EEA665D225C

urn:lsid:ipni.org:names:77362642-1

Figs 13 , 14

##### Type.

Uzbekistan • Jizzakh: northern slopes of the Turkestan range, upper reaches of Sanzar, Guralash-Sai reserve, on the rocky screes of Lyangar-Sai, 14 August 1937, *E.E. Korotkova, A.P. Vasilkovskaya 1024* (holotype: TASH [TASH055342]!).

##### Diagnosis.

*Elymusuzbekistanicus* differs from the morphologically closely similar *E.praeruptus* in the type of pubescence of the spikelet axis – the spikelet axis along the ribs and along the back is usually with either long or short hairs, but is not glabrous or bristly along the ribs (Fig. 15). These two species are similar in blade width 1–4 (5) mm but the leaves of *E.uzbekistanicus* are longitudinally rolled or more or less flat, with long, dense hairs protruding adaxial and abaxial surfaces, in contrast to the leaves of *E.praeruptus*, which are glabrous or covered with very short hairs on the upper surface (Fig. 16).

##### Description.

Herbs, perennial, forming dense turf, without creeping underground shoots, bluish glaucous. Stems erect, 50–100 cm, pubescent with dense, long hairs; nodes densely long-hairy. Sheaths hairy. Ligule 1–1.5 mm. Leaves 1–4 (5) mm wide, grayish green, convolute or more or less flat, both surfaces protruding densely long-hairy. Inflorescence - straight, less often slightly drooping, usually linear, (5) 10–17 (20) cm. Spikelets solitary at each node of axis of spike, all uniform (8) 10–15 (20) mm, with 3–7 bisexual florets, violet; axis of spikelet along ribs and along the back with long hairs, either at the bottom with more or less short hairs. Glumes (4) 5–7 (9) veined; upper glume 8–10 mm, lower glume 9–11 mm. Lemma 8–9 mm, adaxially with dense even spines merging into hairs; awn flexuous, to 25 mm. Palea 7–8 mm. Callus of lemma with few hairs. Anthers 2–3 mm.

##### Distribution and habitat.

*Elymusuzbekistanicus* grows on rocky screes at 2900 m. It is known only from the type locality on northern slopes of the Turkestan Range, Guralash river basin, in Jizzakh province, Uzbekistan (Fig. 17). *Elymusuzbekistanicus* belongs to the Northern Turkestan botanical-geographical region within the Kuhistan botanical-geographical district.

##### Etymology.

The specific epithet refers to the country, Uzbekistan.

##### Phenology.

Flowering and fruiting: most likely in August.

##### Conservation status.

Because the species is known only from the type locality it can be categorized as critically endangered according to IUCN criterion B (e.g. Alam and Ali 2010; Wagensommer et al. 2014, 2017; Le Breton et al. 2019). The restricted range of *E.uzbekistanicus* highlights its conservation significance and underscores the need for further studies on its population status and ecological adaptations.

##### Notes.

Within the genus *Elymus* in Uzbekistan, *E.uzbekistanicus* exhibits a notable morphological distinctiveness, primarily due to its significantly higher pubescence compared to other species. This characteristic is particularly evident in the leaf sheaths, stem, node, and spikelet axis, which are densely covered with hairs, distinguishing it from closely related taxa.

A specimen of *E.uzbekistanicus* in TASH, was identified by V. P. Drobow as *Agropyronugamicum* Drobow, a synonym of *Elymusnevskii* Tzvelev (a quite common and widespread species), first described from the Western Tian Shan (Tzvelev and Probatova 2019). Morphologically, these two species are also similar, sharing such features as straight spikes and lemmas that are abaxially quite densely covered with spines. In *E.nevskii*, however, the axis of the spikelet is rough only along the ribs, and they are abaxially glabrous. The leaf blades are usually glabrous abaxially and the nodes of the culms are always glabrous. The awn of the lemmas is up to 6 mm. Those features are all different from those of *E.uzbekistanicus*, as described herein (Fig. 14). Furthermore, such features of *E.uzbekistanicus* as location of leaf blades on top along thick and strongly projecting ribs and the dense short hairs, or less often spines, suggest that it belongs to sect. Anthosachne (Tzvelev 1976). Both phylogenetically and morphologically *E.uzbekistanicus* is similar to *E.praeruptus*, especially in the following features: grayish green, convoluted, violet-colored glumes and narrow leaf blades, as well as in the erect spikes (less often slightly drooping). The sibling relationships in the evolutionary trees based on the cpDNA genome provide evidence for the morphological similarity. However, the two species differ in the morphology of the lemma, palea, and spikelet axis (Table 1, Figs 15, 16).

**Figure 13.**
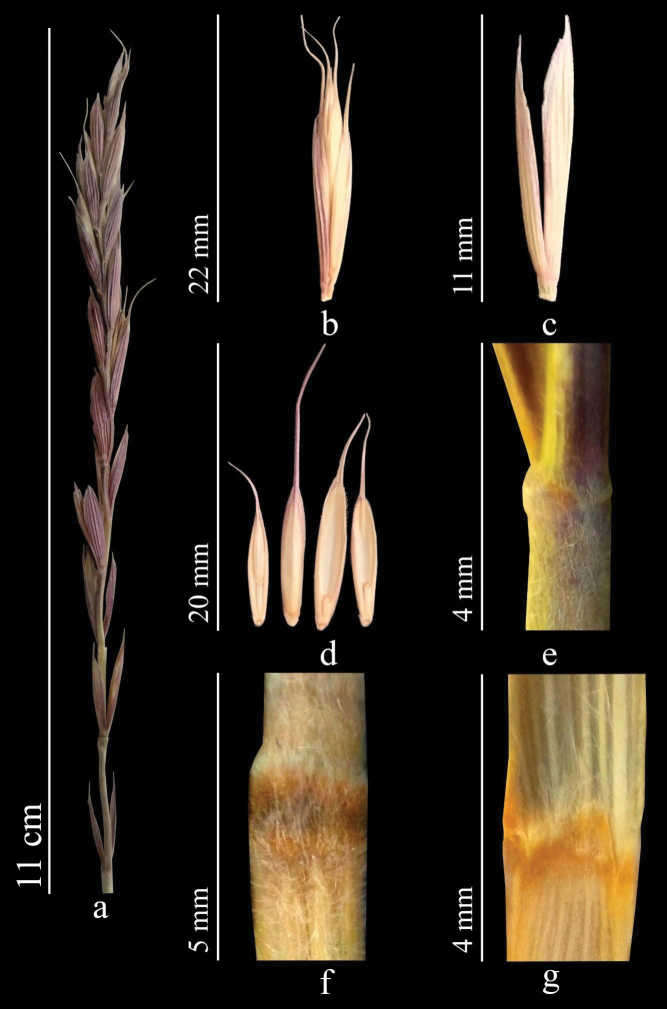
*Elymusuzbekistanicus***a** spike **b** spikelet **c** glumes **d** lemmas and paleas **e** stem **f** node **g** ligule.

**Figure 14.**
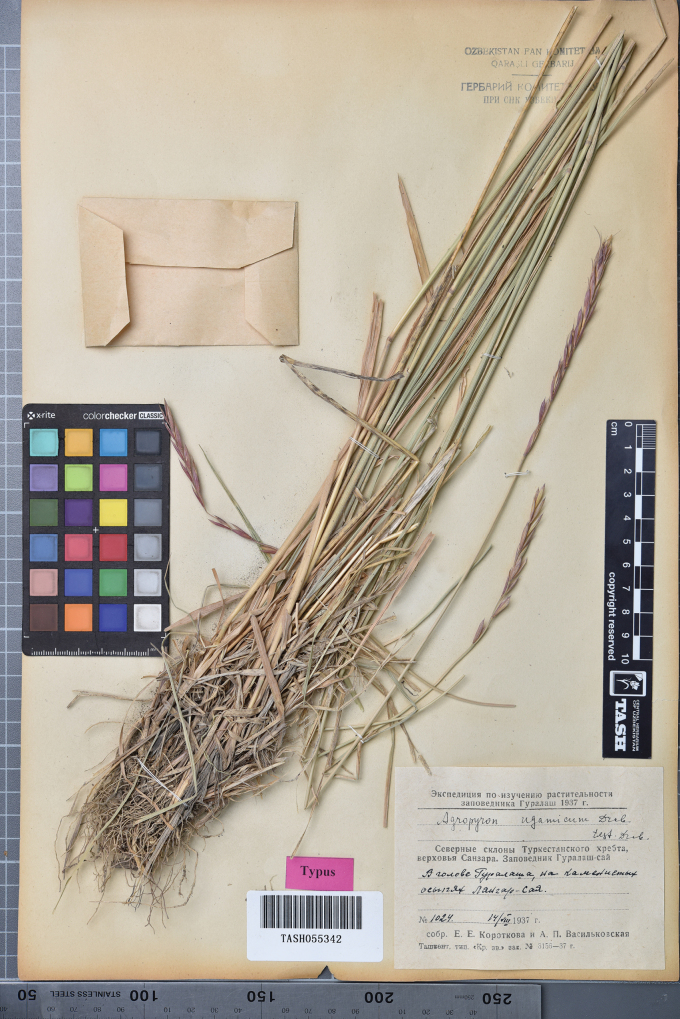
The holotype of *Elymusuzbekistanicus* Usupbaev & Alieva, sp. nov. [TASH055342].

**Figure 15.**
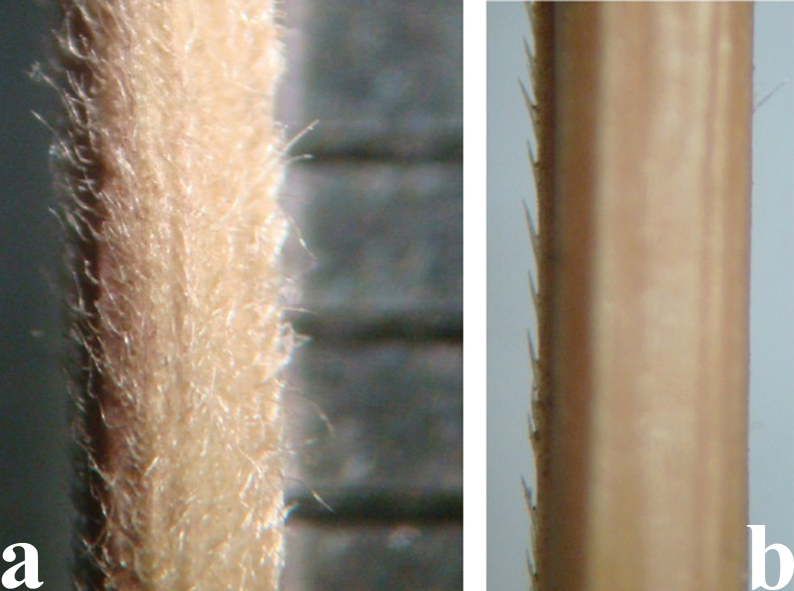
Spikelet axis **a***E.uzbekistanicus* – usually with long or more or less short hairs along the ribs and the back **b***E.praeruptus* – back usually glabrous and smooth; ribs with bristles.

**Figure 16.**
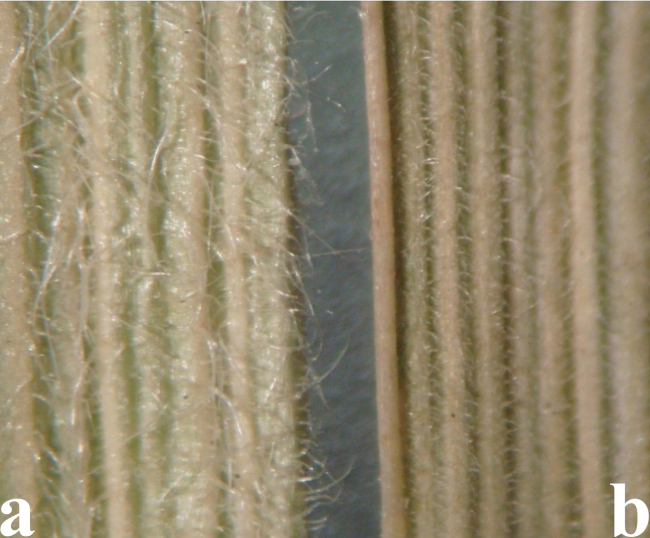
Leaf blades **a***E.uzbekistanicus* – longitudinally rolled or more or less flat, densely covered with long, spreading hairs on both surfaces) **b***E.praeruptus* – adaxial surface covered with short hairs or glabrous.

**Figure 17.**
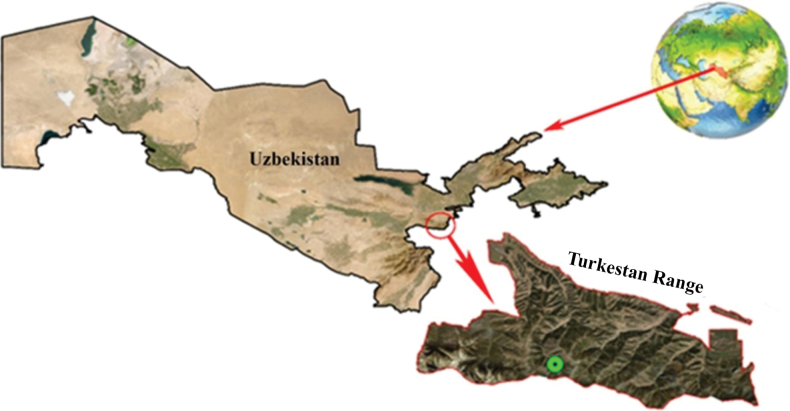
Distribution of *Elymusuzbekistanicus* (Turkestan Range, Uzbekistan)

**Table 1.** Comparison of morphological features of *E.uzbekistanicus*, *E.nevskii*, and *E.praeruptus*.

Features	*E.uzbekistanicus*	*E.nevskii*	*E.praeruptus*
Leaf blades	1–4 (5) mm wide, convolute, more or less flat; densely long-hairy on both surfaces	7–11 mm wide, flat, abaxially glabrous, rough; adaxially sparsely hairy	1.2–4 mm wide, often convolute lengthwise; adaxially densely short hairy and with rather strongly protruding ribs
Spikelet axis	along the ribs and along the back is usually with long hairs, either at the bottom with more or less short hairs	segments rough only along two lateral ribs	lower internodes of axis of rather loose spikes usually smooth or with scattered short spines along the ribs
Lemma	8–9 mm, abaxially with quite dense even spines merging into hairs; awn flexuous, to 25 mm	both surfaces entirely or almost entirely with dense groups of spines or hairs; awn to 7 mm	with scattered spines, often with bluish scurf, 20–30 mm; awn more or less bent to the side
Palea	7–8 mm	linear, almost equal to lemma	linear, almost equal to lemma
Anthers	2–3 mm	1.8-3 mm	3–4.5 mm

**Table 2.** Distribution of species by botanical and geographical regions of Uzbekistan (Tojibaev et al. 2016).

I. Mountain Central Asian province			
**Pamir-Alay Mountain system**			
Botanical-geographical district	Species number	Botanical-geographical region	Species number
I-6 Western Hissar	7	I-6-a Kashkadarya	7
		I-6-c Baysun	3
		I-6-b Tarkapchigay	1
		I-6-d Kuhitang	1
I-5 Kuhistan	6	I-5-a North Turkestan	6
		I-5-b Malguzar	1
I-7 Hissar-Darvaz	5	I-7-a Sangardak-Tupalang	5
I-3 Fergana-Alay	2	I-3-b Eastern Alay	2
**Tian Shan Mountain system**			
I-1 Western Tian Shan	7	I-1-a Ugam-Pskem	6
		I-1-b Western Chatkal	6
		I-1-d Lurama	6
		I-1-c Arashan	3
		I-1-e Chorkesar	1

## ﻿Discussion

Currently, the genus *Elymus* comprises 11 pecies identified in Uzbekistan. According to a previous study (Drobow 1941), a total of 7 species, i.e., *E.giganteus* Vahl, *E.angustus* Trin., *E.angustiformis* Drobow, *E.tianschanicus* Drobow, *E.alaicus* Korsh., *E.ugamicus* Drobow, *E.multicaulis* Kar. & Kir., were now identified as *Leymus* species. As noted in the work of \Nikiforova (1968), in Middle Asia there were 25 described species of *Elymus*, of which only *E.sibiricus* is considered the accepted species of *Elymus* according to the current taxonomy (Govaerts 2024), but this species does not occur in Uzbekistan. In reference to the POWO website, 12 species of the genus are distributed in the flora of Uzbekistan. However, *E.lolioides* (P. Candargy) Melderis and *E.repens* (L.) Gould, which are listed in this database, have been classified by Tzvelev and Probatova (2019) under the genus ElytrigiaDesv.,sect.Elytrigia, as *Elytrigialolioides* (Kar. et Kir.) Nevski and *Elytrigiarepens* (L.) Nevski. This classification is based on morphological characteristics, such as the presence of either tufted growth or long creeping rhizomes. In addition, *E.czimganicus* (Drobow) Tzvelev was included in the flora list under the name *E.tschimganicus* (Drobow) Tzvelev by Tzvelev (1976). Additionally, the POWO website lists *E.uralensis* (Nevski) Tzvelev as part of the flora of Uzbekistan. However, Tzvelev and Probatova (2019) classified this species as endemic to the Southern Ural region. Tzvelev (1976) recorded E.uralensissubsp.tianschanicus (Drobow) Tzvelev in the Western Tien Shan (Khumsan, Haudale Mountains). This taxon was later classified by Cherepanov (1981) as *E.tianschanigenus* Czer. Moreover, as reported by the POWO website, *E.dentatus* (Hook.f) Tzvelev is listed as a species belonging to the flora of Uzbekistan. Subdivisions of this species were classified by Tzvelev (1976) as subspecies, namely E.dentatussubsp.ugamicus (Drobow) Tzvelev and E.dentatussubsp.lachnophyllus (Ovcz. et Sidor.) Tzvelev. However, Cherepanov (1981) reclassified E.dentatussubsp.lachnophyllus as *E.lachnophyllus* (Ovcz. et Sidor.) Tzvelev. Later, Tzvelev and Probatova (2019) reclassified E.dentatussubsp.ugamicus as *E.nevskii* Tzvelev. Finally, the revision of the herbarium specimens, stored in MW, US, W, TASH, and SAMDU led us to exclude *E.gmelinii* (Trin.) Tzvelev and *E.abolinii* (Drobow) Tzvelev from the flora of Uzbekistan.

Our analyses shows that the ITS and plastome trees generated inconsistent topologies between the clades. We speculated that the reason for these discrepancies may be different inheritance patterns because the nuclear and plastid genomes are inherited through different mechanisms, which is that the nuclear genome is typically biparentally inherited, whereas the plastid genome is maternally inherited in most plants. These distinct inheritance patterns can lead to incongruence in the phylogenetic signals (Birky 2001; Petit et al. 2004; Wicke et al. 2011). The second possibility is that the differential rates of evolution mean that the nuclear and plastid genomes evolve independently, especially that the nuclear genome generally evolves more slowly than the plastid genome, and that such rate differences can affect the resolution and placement of species in phylogenetic trees (Palmer 1985; Wolfe et al. 1987; Koch et al. 2001). In addition, it was proposed that incomplete lineage sorting (ILS) and hybridization might be the reasons for these discrepancies between ITS and plastome-based phylogenies (Pelser et al. 2010; Suh et al. 2015). In fact, hybridization and ILS are prevalent in the Poaceae, for example in bamboo (Bambusoideae Luerss.), wheat (*Triticum* L.), rye (*Secale* L.) or feathergrasses (*Stipa* L.; (Feldman and Levy 2012; Kelchner et al. 2013; Nobis et al. 2019; Baiakhmetov et al. 2021; Sinaga et al. 2024). The evolutionary history of the Poaceae has been determined by hybridization and ILS, which have impacted the evolution of numerous plant lineages within the family, according to molecular phylogenetic research (Feldman and Levy 2009; Edwards et al. 2010; Kelchner et al. 2013). The main causes of differences between phylogenetic reconstructions of species of *Elymus* based on nuclear and plastid DNA data are intricate evolutionary processes, such as hybridization, allopolyploidy, and imperfect lineage sorting. The mixing of genomes from various species, known as allopolyploidy, can cause nuclear and plastid phylogenies to be incongruent because of the varied inheritance patterns of these genomes. Phylogenetic investigations are made more difficult by hybridization events, which introduce genetic material from several lineages. Another factor contributing to such disparities is incomplete lineage sorting, in which ancestral genetic variations are maintained differently in descendent species. St genome of *Pseudoroegneria* has been shown to be a shared maternal ancestor of *Elymus*, suggesting a complex reticulate evolution (Dong et al. 2015). Furthermore, it has been proposed that incongruences between nuclear ribosomal DNA sequences and plastid DNA sequences can be resolved by using single-copy nuclear genes (Mason-Gamer et al. 2010). To provide additional understanding of the evolutionary relationships between these taxa, further, more advanced molecular studies are needed.

## Supplementary Material

XML Treatment for Elymus sect. Anthosachne

XML Treatment for Elymus sect. Goulardia

XML Treatment for Elymus caninus

XML Treatment for Elymus fedtschenkoi

XML Treatment for Elymus lachnophyllus

XML Treatment for Elymus longe-aristatus

XML Treatment for Elymus macrochaetus

XML Treatment for Elymus nevskii

XML Treatment for Elymus praeruptus

XML Treatment for Elymus tianschanigenus

XML Treatment for Elymus transhyrcanus

XML Treatment for Elymus tschimganicus

XML Treatment for Elymus uzbekistanicus

## ﻿Additional information

### Author ORCIDs

Kumush B. Alieva https://orcid.org/0000-0002-8795-3749

Adilet Usupbaev https://orcid.org/0000-0002-6503-9761

Komiljon Sh. Tojibaev https://orcid.org/0000-0003-2846-5777

Ziyoviddin Yusupov https://orcid.org/0000-0003-2278-542X

Ibrokhimjon Ergashov https://orcid.org/0000-0002-0991-1076

Dilnoza Azimova https://orcid.org/0000-0003-1573-1095

Zhilin Jiang https://orcid.org/0000-0003-4501-1953

### Data availability

All of the data that support the findings of this study are available in the main text.

## ﻿Appendix 1

**Table A1.** The GenBank accession and voucher information for the species utilized in our research. An asterisk indicates new sequences generated during this study (*).

Species	Voucher specimens for the herbarium	GenBank’s accession number
		nrDNA (ITS)/ cpDNA
*Bromusinermis* Leyss.	Turkey, Ilhan Kaya & Muzaffer Mukemre *s.n.*^1^	MW270937^1^/ MW861351
*Elymuscaninus* (L.) L.*	Uzbekistan, V. S. Titov & Ye. Ye. Korotkova *TASH040338* (TASH)	PQ222997/ PQ380098
*Elymusfedtschenkoi* Tzvelev*	Uzbekistan, D. Turdiev & K. Alieva *TASH054578* (TASH)	PQ223000/ PQ385828
*Elymuslachnophyllus* (Ovcz. & Sidorenko) Tzvelev*	Uzbekistan, N. Koshurnikova *TASH046021* (TASH)	PQ301177/PQ380099
*Elymuslongearistatus* (Boiss.) Tzvelev*	Uzbekistan, F. O. Khasanov *TASH126639* (TASH)	PQ222996/ PQ385829
*Elymusmacrochaetus* (Nevski) Tzvelev*	Uzbekistan, O. Khasanov *TASH041091* (TASH)	PQ222999/ PQ385830
*Elymusnevskii* Tzvelev*	Uzbekistan, Savich *TASH046204* (TASH)^1^	PQ223001^1^/PQ385831^1^, PQ450443^2^
	Uzbekistan, D. Turdiev & K. Alieva *TASH054576* (TASH)^2^	
*Elymuspraeruptus* Tzvelev*	Uzbekistan, D. Turdiev & K. Alieva *TASH00246764* (TASH)^1^	PV022377^1^/PV054326^1^PQ385832^2^
	Uzbekistan, K. Tojibaev & I. Juramurodov *TASH060159* (TASH)^2^	
*Elymustianschanigenus* Czerep.	Uzbekistan, Z. Maylun, M. Nabiyev, T. Tsukervanik *TASH043726* (TASH)	PV036609/PV054327
*Elymustranshyrcanus* (Nevski) Tzvelev*	Uzbekistan, Nabiev & Li *TASH047580* (TASH)^1^	PQ223004^1^/ PQ450445^2^
	Uzbekistan, D. Turdiev & K. Alieva *TASH054580* (TASH)^2^	
*Elymustschimganicus* (Drobow) Tzvelev*	Uzbekistan, V. Bochantsev & A. Butkov *TASH040994* (TASH)	PQ223008/PQ450446
*Elymusuzbekistanicus* Usupbaev & Alieva*	Uzbekistan, E. Korotkova & A. Vasilkovskaya *TASH055342*	PQ227098/PQ362383
*Elymusantiquus* (Nevski) Tzvelev	China, *H3400* (H)^1^	AY740819^1^, KF905163^2^/ MT089577^3^. NC_059974^3^
	China, *EI_201219*^2^	
	US, *PI 632564*^3^	
*Elymusatratus* (Nevski) Hand.-Mazz.	China, *SAUTI ZY 3023*^1^	KJ526331^1^/ MT610373^2^, NC_061050^2^
	China, *CY201201*^2^	
*Elymusciliaris* (Trin.) Tzvelev	China, *H7000*^1^	AY740831^1^, KF713219, KF713221, MH808818^2^/ MK775252^3^
	South Korea: Icheon, *HCCN-PJ008548-PB-65*^2^	
	China, *s.n*^3^	
*Elymushystrix* L.	China, *PI372546*^1^	EF396973^1^/ MT106331^2^, NC_058749^2^
	China, *PI 372546*^2^	
*Elymusvirginicus* L.	China, *PI583297*^1^	FJ040170^1^/ MT106332^2^, NC_058750^2^
	China, *PI 883297*^2^	

## References

[B1] Alam J Ali SI (2010) Contribution to the red list of the plants of Pakistan. Pakistan Journal of Botany 42(5): 2967–2971.

[B2] Alieva K (2023) *Elymus*. Institute of Botany of the Academy of Sciences of the Republic of Uzbekistan. Occurrence dataset 10.15468/eu2rfj [accessed via GBIF.org on 2025-04-24]

[B3] Alieva K Tojibaev K Karimov F (2024a) *Elymus* L. in the flora of Uzbekistan. Version 1.23. Institute of Botany of the Academy of Sciences of the Republic of Uzbekistan. Occurrence dataset. 10.15468/y5b8hu [accessed via GBIF.org on 2025-04-24]

[B4] Alieva KB Sheraliev O Uralov R Djoldoshbekov AD Usupbaev AK Tojibaev KSh (2024b) New findings of grasses for the flora of Uzbekistan and Kyrgyzstan. Turczaninowia 27(4): 45–60. 10.14258/turczaninowia.27.4.6

[B5] Andrés Sánchez S Lucía V Martínez Ortega MM Rico E (2021) (2839) Proposal to conserve the name *Triticumcaninum* (*Elymuscaninus*) (Poaceae) with a conserved type. Taxon 70(5): 1138–1139. 10.1002/tax.12582

[B6] Baiakhmetov E Ryzhakova D Gudkova PD Nobis M (2021) Evidence for extensive hybridisation and past introgression events in feather grasses using genome-wide SNP genotyping. BMC Plant Biology 21(1): 505. 10.1186/s12870-021-03287-w 34724894 PMC8559405

[B7] Barkworth M Burkhamer R Talbert L (1996) *Elymuscalderi*: A new species in the Triticeae (Poaceae). Systematic Botany 21(3): 349–354. 10.2307/2419663

[B8] Birky Jr CW (2001) The inheritance of genes in mitochondria and chloroplasts: Laws, mechanisms, and models. Annual Review of Genetics 35(1): 125–148. 10.1146/annurev.genet.35.102401.090231 11700280

[B9] Bor NL (1970) Gramineae. Flora Iranica 70/30. Akademische Druck-und Verlagsanstalt, 1–573.

[B10] Cherepanov SK (1981) Vascular Plants of Russia and Neighboring States (within the Former USSR). Mir I Semya, Sankt Petersburg, 992 pp.

[B11] Dierckxsens N Mardulyn P Smits G (2017) NOVOPlasty: De novo assembly of organelle genomes from whole genome data. Nucleic Acids Research 45: 18. 10.1093/nar/gkw955 PMC5389512 28204566

[B12] Dong ZZ Fan X Sha LN Wang Y Zeng J Kang HY Zhang HQ Wang XL Zhang L Ding CB Yang R-W Zhou Y-H (2015) Phylogeny and differentiation of the St genome in *Elymus* L. sensu lato (Triticeae; Poaceae) based on one nuclear DNA and two chloroplast genes. BMC Plant Biology 15(1): 1–14. 10.1186/s12870-015-0517-2 26164196 PMC4499217

[B13] Drobow V (1925) Gramineae novae turkestanicae. I. Repertorium novarum Specierum Regni Vegetabilis 21(1–7): 37–46. 10.1002/fedr.19250210106

[B14] Drobow VP (1941) *Elymus* L. In: Kudrjaschev SN (Eds) Flora of Uzbekistan.Fan Press, Tashkent 1: 302–305. [In Russian]

[B15] Edler D Klein J Antonelli A Silvestro D (2020) raxmlGUI 2.0: A graphical interface and toolkit for phylogenetic analyses using RAxML. Methods in Ecology and Evolution 12(2): 373–377. 10.1111/2041-210X.13512

[B16] Edwards EJ Osborne CP Strömberg CAE Smith SA Bond WJ Christin P-A Cousins AB Duvall MR Fox DL Freckleton RP Ghannoum O Hartwell J Huang Y Janis CM Keeley JE Kellogg EA Knapp AK Leakey ADB Nelson DM Saarela JM Sage RF Sala OE Salamin N Still CJ Tipple B (2010) The origins of C4 grasslands: Integrating evolutionary and ecosystem science. Science 328(5978): 587–591. 10.1126/science.1177216 20431008

[B17] Fedtschenko BA (1932) Genus (70) *Elymus* L. In: Fedtschenko BA Popova MG (Eds) Flora of Turkmenistan.An SSSR, Leningrad 1: 207–212.

[B18] Feldman M Levy AA (2009) Genome evolution in allopolyploid wheat—A revolutionary reprogramming followed by gradual changes. Journal of Genetics and Genomics 39(9): 511–518. 10.1016/S1673-8527(08)60142-3 19782952

[B19] Feldman M Levy AA (2012) Genome evolution due to allopolyploidization in wheat. Genetics 192(3): 763–774. 10.1534/genetics.112.146316 23135324 PMC3522158

[B20] Frawley E Ciotir C Micke B Rubin M Miller A (2019) An ethnobotanical study of the genus *Elymus*. 40 pp. 10.1101/734525

[B21] Gaem PH Andrella GC Maurin O Bittrich V Mazine FF Lucas E Amaral MC (2024) Integrating datasets from herbarium specimens and images to treat a Neotropical myrtle species complex. Annals of Botany XX: 1–17. 10.1093/aob/mcae183 PMC12259539 39431944

[B22] German D Madaminov F Beshko N (2024) A new, highly endangered and restricted-range species of Parryasect.Pseudoclausia, comb. nov. (Brassicaceae) from Western Tian Shan, Uzbekistan. Phytotaxa 633(2): 145–154. 10.11646/phytotaxa.633.2.5

[B23] Govaerts R [Ed.] (2024) WCVP: World Checklist of Vascular Plants. Facilitated by the Royal Botanic Gardens, Kew. [WWW document] http://sftp.kew.org/pub/data-repositories/WCVP/ [accessed 21 May 2024]

[B24] Huelsenbeck JP Ronquist F (2001) MRBAYES: Bayesian inference of phylogenetic trees. Bioinformatics 17(8): 754–755. 10.1093/bioinformatics/17.8.754 11524383

[B25] Jacobs S Barkworth M (2009) A New Species of *Elymus* (Gramineae, Triticeae) from Eastern Australia. Novon 19(2): 168–171. 10.3417/2008094

[B26] Jin JJ Yu WB Yang JB Song Y dePamphilis CW Yi T-S Li D-Z (2020) GetOrganelle: A fast and versatile toolkit for accurate de novo assembly of organelle genomes. Genome Biology 21(1): 1–31. 10.1186/s13059-020-02154-5 PMC7488116 32912315

[B27] Juramurodov I Tojibaev K Nikitina V Makhmudjanov D Yusupov Z Deng T Dekhkonov D (2021) *Hedysarumsunhangii* (Fabaceae, Hedysareae), a new species from Pamir-Alay (Babatag Ridge - Uzbekistan). Phytotaxa 524(1): 1–13. 10.11646/phytotaxa.524.1.1

[B28] Juramurodov I Tojibaev K Sennikov A Makhmudjanov D (2024) The genus *Hedysarum* (Fabaceae; Hedysareae) in Uzbekistan. Plant Diversity of Central Asia 3(1): 1–90. 10.54981/PDCA/vol3_iss1/a

[B29] Katoh K Standley DM (2013) MAFFT multiple sequence alignment software version 7: Improvements in performance and usability. Molecular Biology and Evolution 30(4): 772–780. 10.1093/molbev/mst010 23329690 PMC3603318

[B30] Kearse M Moir R Wilson A Stones-Havas S Cheung M Sturrock S Buxton S Cooper A Markowitz S Duran C Thierer T Ashton B Meintjes P Drummond A (2012) Geneious Basic: An integrated and extendable desktop software platform for the organization and analysis of sequence data. Bioinformatics 28(12): 1647–1649. 10.1093/bioinformatics/bts199 22543367 PMC3371832

[B31] Kelchner SA (2013) Higher-level phylogenetic relationships within the bamboos (Poaceae: Bambusoideae) based on multilocus plastid and nuclear sequences. Molecular Phylogenetics and Evolution 67: 404–413. 10.1016/j.ympev.2013.02.005 23454093

[B32] Khassanov F Pulatov S Asatulloev T Ergashov I Tojibaev K Yusupov Z (2023) *Alliumsunhangii* -a new species from section Brevidentia F.O.Khass. & Iengal. (Amaryllidaceae) from Southern Pamir-Alay, Uzbekistan. PhytoKeys 219: 35–48. 10.3897/phytokeys.219.96464 37252455 PMC10209640

[B33] Koch M Haubold B Mitchell-Olds T (2001) Molecular systematics of the Brassicaceae: Evidence from coding plastidic *mat*K and nuclear *Chs* sequences. American Journal of Botany 88(3): 534–544. 10.2307/2657117 11250830

[B34] Kumar S Stecher G Li M Knyaz C Tamura K (2018) MEGA X: Molecular evolutionary genetics analysis across computing platforms. Molecular Biology and Evolution 35(6): 1547–1549. 10.1093/molbev/msy096 29722887 PMC5967553

[B35] Kuznetsov NM (1956) *Elymus* L. In: Pavlov NV (Ed.) Flora Kazakhstana.An Kazakhsk SSR Press, Alma-Ata 1: 318–329. [In Russian]

[B36] Le Breton TD Zimmer HC Gallagher RV Cox M Allen S Auld TD (2019) Using IUCN criteria to perform rapid assessments of at-risk taxa. Biodiversity and Conservation 28(4): 863–883. 10.1007/s10531-019-01697-9

[B37] Levichev IG Karimov FI Kurbaniyazova GT (2025) A new species of Gagea (Liliaceae) from the Ferghana Valley (Uzbekistan). Turczaninowia 28(1): 95–101. 10.14258/turczaninowia.28.1.11 PMC12322680 40765898

[B38] Lu BR (1994) The genus *Elymus* in Asia. Taxonomy and biosystematics with special reference to genomic relationships. In: Wang RRC Jensen K (Eds) Proceedings of the 2nd international Triticeae symposium.Logan, Utah, USA, 219–233.

[B39] Makhmudjanov D Juramurodov I Kurbonalieva M Yusupov Z Dekhkonov D Deng T Tojibaev K Sun H (2022) Genus *Eremurus* (Asphodelaceae) in the flora of Uzbekistan. Plant Diversity of Central Asia 1(2): 82–127. 10.54981/PDCA/vol1_iss2/a4

[B40] Mason-Gamer RJ Burns MM Naum M (2010) Reticulate Evolutionary History of a Complex Group of Grasses: Phylogeny of *Elymus* StStHH Allotetraploids Based on Three Nuclear Genes. PLoS ONE 5(6): e10989. 10.1371/journal.pone.0010989 PMC2882950 20543952

[B41] Nikiforova NB (1968) Genus *Elymus* L. In: Vvedenskiy AI (Eds) Conspectus Florae Asiae Mediae.Fan Press, Tashkent 1: 188–197.

[B42] Nobis M Gudkova PD Baiakhmetov E Żabicka J Krawczyk K Sawicki J (2019) Hybridisation, introgression events and cryptic speciation in Stipa (Poaceae): A case study of the Stipa heptapotamica hybrid-complex. Perspectives in Plant Ecology, Evolution and Systematics 39: 125457. 10.1016/j.ppees.2019.05.001

[B43] Nobis M Gudkova P Nowak A Sawicki J Nobis A (2020) A synopsis of the genus Stipa (Poaceae) in Middle Asia, including a key to species identification, an annotated checklist, and phytogeographic analyses. Annals of the Missouri Botanical Garden 105(1): 1–63. 10.3417/2019378

[B44] Palmer JD (1985) Comparative organization of chloroplast genomes. Annual Review of Genetics 19(1): 325–354. 10.1146/annurev.ge.19.120185.001545 3936406

[B45] Patel RK Jain M (2012) NGS QC Toolkit: A toolkit for quality control of next generation sequencing data. PLoS ONE 7(2): 30619. 10.1371/journal.pone.0030619 PMC3270013 22312429

[B46] Pelser PB Kennedy AH Tepe EJ Shidler JB Nordenstam B Kadereit JW Watson LE (2010) Patterns and causes of incongruence between plastid and nuclear Senecioneae (Asteraceae) phylogenies. American Journal of Botany 97(5): 856–873. 10.3732/ajb.0900287 21622451

[B47] Petit RJ Duminil J Fineschi S Hampe A Salvini D Vendramin GG (2004) Invited review: Comparative organization of chloroplast, mitochondrial and nuclear diversity in plant populations. Molecular Ecology 14(3): 689–701. 10.1111/j.1365-294X.2004.02410.x 15723661

[B48] Pimenov M Tojibaev K Degtjareva G Ostroumova T Zakharova E Karimov F Samigullin T (2023) A new species of *Aulacospermum* (Umbelliferae) from Uzbekistan. Phytotaxa 579(3): 162–174. 10.11646/phytotaxa.579.3.2

[B49] POWO (2025) Plants of the World Online. Facilitated by the Royal Botanic Gardens, Kew. https://powo.science.kew.org/ [Retrieved 28 March 2025]

[B50] Pusalkar P Singh D Srivastava S (2008) *Elymusgangotrianus* (Poaceae: Pooideae: Triticeae), a new species from the western Himalaya, India. Kew Bulletin 63(3): 507–509. 10.1007/s12225-008-9046-6

[B51] Rambaut A (2012) FigTree v1. 4.0. University of Oxford, Oxford, UK. http://tree.bio.ed.ac.uk/software/figtree [Accessed 28.10.2021]

[B52] Rozhevits RYu (1950) Genus (70) *Elymus* L. In: Shishkin BK (Eds) Flora of the Kirghiz SSR.Frunze, Bishkek 2: 217–222.

[B53] Salomon BA (1990) New Species of *Elymus* (Poaceae) from China. Willdenowia 19(2): 449–451. http://www.jstor.org/stable/3996653

[B54] Schreder RR Korovin EP [Eds] (1941) Flora of Uzbekistan, Vol. 1. Academy of Sciences of the Uzbek SSR, Tashkent, 566 pp. [In Russian]

[B55] Sennikov AN [Ed.] (2016) Flora of Uzbekistan, Vol. 1. Navro‛z Publishers, Tashkent [xxviii +] 173 pp. [In Russian]

[B56] Sennikov AN [Ed.] (2017) Flora of Uzbekistan, Vol. 2. Navro‛z Publishers, Tashkent [xii +] 200 pp. [In Russian]

[B57] Sennikov AN [Ed.] (2019) Flora of Uzbekistan, Vol. 3. Ma’naviat Publishers, Tashkent [xii +] 201 pp. [In Russian]

[B58] Sennikov AN [Ed.] (2022) Flora of Uzbekistan, Vol. 4. AS RUz “Fan” Publishers, Tashkent [xviii +] 238 pp. [In Russian]

[B59] Sennikov AN [Ed.] (2023a) Flora of Uzbekistan, Vol. 5. AS RUz “Fan” Publishers, Tashkent [xviii +] 238 pp. [In Russian]

[B60] Sennikov AN [Ed.] (2023b) Flora of Uzbekistan, Vol. 6. “Ma’naviyat” Publishers, Tashkent [xviv +] 209 pp. [In Russian]

[B61] Sennikov AN Tojibaev KS Khassanov FO Beshko NY (2016) The flora of Uzbekistan project. Phytotaxa 282(2): 107–118. 10.11646/phytotaxa.282.2.2

[B62] Sennikov A Khassanov F Pulatov S (2022) *Irisbucharica* (Iridaceae): A century of confusion is resolved with the description of *I.chrysopetala*, a new species from southern Central Asia. Memoranda Societatis Pro Fauna et Flora Fennica 98: 9–20.

[B63] Sennikov A Khassanov F Ortikov E Kurbonaliyeva M Tojibaev KSh (2023) The genus *Iris* L. sl (Iridaceae) in the Mountains of Central Asia biodiversity hotspot. Plant Diversity of Central Asia 2(1): 1–104. 10.54981/PDCA/vol2_iss1/a1

[B64] Shchegoleva NV Nikitina V Juramurodov I Zverev A Turginov OT Jabborov A Yusupov Z Dekhkonov D Deng T Sun H (2022) A new species of *Ranunculus* (Ranunculaceae) from Western Pamir-Alay, Uzbekistan Launched to accelerate biodiversity research. PhytoKeys 193: 125–139. 10.3897/phytokeys.193.70757 35586122 PMC9005467

[B65] Sidorenko GT (1957) *Elymus* L. In: Ovchinnikov PN (Eds) Flora of the Tajikistan SSR.Academy of Sciences of the USSR, Nauka Press Moskva-Leningrad 1: 268–272.

[B66] Sinaga P Klichowska E Nowak A Nobis M (2024) Hybridization and introgression events in cooccurring populations of closely related grasses (Poaceae: Stipa) in high mountain steppes of Central Asia. PLoS ONE 19(2): e0298760. 10.1371/journal.pone.0298760 PMC10898772 38412151

[B67] Soreng RJ Peterson PM Romaschenko K Davidse G Teisher JK Clark LG Zuloaga FO (2017) A worldwide phylogenetic classification of the Poaceae (Gramineae) II: An update and a comparison of two 2015 classifications. Journal of Systematics and Evolution 55(4): 259–290. 10.1111/jse.12262

[B68] Soreng RJ Peterson PM Zuloaga FO Romaschenko K Clark LG Teisher JK Gillespie LJ Barberá P Welker CA Kellogg EA Li DZ Davidse G (2022) A worldwide phylogenetic classification of the Poaceae (Gramineae) III: An update. Journal of Systematics and Evolution 60(3): 476–521. 10.1111/jse.12847

[B69] Suh A Smeds L Ellegren H (2015) The dynamics of incomplete lineage sorting across the ancient adaptive radiation of neoavian birds. PLoS Biology 13(8): e1002224. 10.1371/journal.pbio.1002224 PMC4540587 26284513

[B70] Swofford D (2002) PAUP*. Phylogenetic Analysis Using Parsimony (*and Other Methods). Version 4.0b10. 10.1111/j.0014-3820.2002.tb00191.x

[B71] Takhtajan AL (1986) Floristic regions of the world. University of California Press, Berkeley, 522 pp.

[B72] Thiers B (2021) Index Herbariorum: A global directory of public herbaria and associated staff. New York Botanical Garden’s Virtual Herbarium. http://sweetgum.nybg.org/science/ih/ [Accessed 4.10.2021]

[B73] Tojibaev KSh Beshko NYu Popov VA (2016) Botanical-geographical regionalization of Uzbekistan. Botanicheskii Zhurnal 101(10): 1105–1132.

[B74] Tojibaev KSh Khassanov FO Beshko NY Tajetdinova DM Turginov OT Sennikov AN Chang KS Oh S Jang C (2019) Diversity and distribution of the genus *Scrophularia* L. (Scrophulariaceae) in uzbekistan. Journal of Asia-Pacific Biodiversity 13(2020): 70–91. 10.1016/j.japb.2019.10.004

[B75] Tojibaev K Dekhkonov D Ergashov I Sun H Deng T Yusupov Z (2022) The synopsis of the genus *Tulipa* (Liliaceae) in Uzbekistan. Phytotaxa 573(2): 163–214. 10.11646/phytotaxa.573.2.2

[B76] Turdiboev O Shormanova AA Sheludyakova MB Akbarov F Drew BT Celep F (2022) Synopsis of the Central Asian *Salvia* species with identification key. Phytotaxa 543(1). 10.11646/phytotaxa.543.1.1

[B77] Tzvelev NN (1968) Rasteniya Tsentralnoy Azii. Leningrad 4: 210–223. 10.3176/lu.1968.3.12

[B78] Tzvelev NN (1970) Spisok rasteniy Gerbariya flory SSSR izdavaemogo Botanicheskim institutom im. V.L. Komarova Akademii nauk SSSR. Leningrad 18: 27–29.

[B79] Tzvelev NN (1972) New taxons of the Poaceae from the flora of the USSR. In: Vasilchenko IT (Ed.) Novosti sistematiki vysshix rasteniy.Nauka Press, Leningrad 9: 55–63.

[B80] Tzvelev NN (1973) Review of species of the Triticeae tribe of the grass family (Poaceae) in the flora of the USSR. In: Vasilchenko IT (Ed.) Novosti sistematiki vysshix rasteniy.Nauka Press, Leningrad 10: 19–56.

[B81] Tzvelev NN (1976) Zlaki SSSR. Nauka Press, Leningrad, 104–127.

[B82] Tzvelev NN Probatova NS (2019) Zlaki Rosii. Moskva, 646.

[B83] Vvedensky AI [Ed.] (1959) Flora of Uzbekistan, Vol. 4. Academy of Sciences of the Uzbek SSR, Tashkent, 507 pp. [In Russian]

[B84] Vvedensky AI [Ed.] (1961) Flora of Uzbekistan, Vol. 5. Academy of Sciences of the Uzbek SSR, Tashkent, 667 pp. [In Russian]

[B85] Vvedensky AI [Ed.] (1962) Flora of Uzbekistan, Vol. 6. Academy of Sciences of the Uzbek SSR, Tashkent, 630 pp. [In Russian]

[B86] Vvedensky AI Korovin EP [Eds] (1953) Flora of Uzbekistan, vol. 2. Academy of Sciences of the Uzbek SSR, Tashkent, 548 pp. [In Russian]

[B87] Vvedensky AI Korovin EP [Eds] (1955) Flora of Uzbekistan, vol. 3. Academy of Sciences of the Uzbek SSR, Tashkent, 825 pp. [In Russian]

[B88] Wagensommer RP Perrino EV Silletti GN (2014) *Carexphyllostachys* C.A. Mey. (Cyperaceae) new for Italy and phytogeographical considerations. Phyton (Horn, Austria) 54(2): 215–222. 10.12905/0380.phyton54(2)2014-0215

[B89] Wagensommer RP Bartolucci F Fiorentino M Licht W Peccenini S Perrino EV Venanzoni R (2017) First record for the flora of Italy and lectotypification of the name *Linumelegans* (Linaceae). Phytotaxa 296(2): 161–170. 10.11646/phytotaxa.296.2.5

[B90] Wicke S Schneeweiss GM Depamphilis CW Müller KF Quandt D (2011) The evolution of the plastid chromosome in land plants: Gene content, gene order, gene function. Plant Molecular Biology 76(3–5): 273–297. 10.1007/s11103-011-9762-4 21424877 PMC3104136

[B91] Wolfe KH Li WH Sharp PM (1987) Rates of nucleotide substitution vary greatly among plant mitochondrial, chloroplast, and nuclear DNAs. Proceedings of the National Academy of Sciences of the United States of America 84(24): 9054–9058. 10.1073/pnas.84.24.9054 3480529 PMC299690

[B92] Xiong Y Xiong Y Jia X Zhao J Yan L Sha L Liu L Yu Q Lei X Bai Sh Ma X (2023) Divergence in *Elymussibiricus* is related to geography and climate oscillation: A new look from pan‐chloroplast genome data. Journal of Systematics and Evolution 62(4): 794–808. 10.1111/jse.13020

[B93] Yadav R Agnihotri P Madhukar V Husain T (2023) *Elymusnepalensis* (Poaceae, Triticeae): A rediscovery, lectotypification, and new geographic record from India. Feddes Repertorium 134(2): 89–93. 10.1002/fedr.202200044

